# Toxic effects of *Helix aspersa* snail egg hydrolyzates obtained by static *in vitro* digestion on Caco-2 colorectal adenocarcinoma cells

**DOI:** 10.1038/s41598-025-11605-7

**Published:** 2025-07-25

**Authors:** Magdalena Matusiewicz, Joanna Kuczka, Michalina Danił, Klara Piotrowska, Hanna Antushevich, Tomasz Niemiec

**Affiliations:** 1 Department of Nanobiotechnology, Institute of Biology, Warsaw University of Life Sciences, Warsaw, Poland; 2 Department of Animal Breeding and Nutrition, Institute of Animal Sciences, Warsaw University of Life Sciences, Warsaw, Poland; 3 Department of Genetic Engineering, The Kielanowski Institute of Animal Physiology and Nutrition, Polish Academy of Sciences, Jabłonna, Poland

**Keywords:** Biochemistry, Biological techniques, Biotechnology, Cancer, Cell biology, Drug discovery, Molecular biology, Physiology, Diseases, Gastroenterology, Medical research, Oncology

## Abstract

Colorectal cancer is the third most common cancer. Anticancer compounds obtained after the digestion of snail eggs may hurt the growth and development of colon cancer cells. The research aimed to prepare hydrolyzates from *Helix aspersa maxima* and *Helix aspersa aspersa* snail eggs using static *in vitro* simulation of gastrointestinal digestion. Redox status indicators, concentration of total protein, total carbohydrates, uronic acids, allantoin, and glycolic acid, and electrophoretic profile of proteins and glycoproteins were compared in hydrolyzates and non-digested extracts. The effect of hydrolyzates on the integrity of the plasma membrane of Caco-2 human epithelial colorectal adenocarcinoma cells and the monolayer of IEC-6 rat intestinal cells was determined. The influence of hydrolyzates on the production of reactive oxygen species (ROS) by Caco-2 cells, the potential of their mitochondrial membrane, and the production of apoptotic proteins were examined. Incubation with hydrolyzates for 72 h reduced the plasma membrane integrity of Caco-2 cells but not normal cells, did not affect ROS content, decreased mitochondrial membrane potential (the tendency for *H. a. aspersa* hydrolyzate), and altered the concentration of some apoptotic proteins of intrinsic and extrinsic apoptosis pathways. Bioactive compounds released during digestion and their interactions may have been responsible for anticancer effects.

**Supplementary Information:**

The online version contains supplementary material available at 10.1038/s41598-025-11605-7.

## Introduction

Colorectal cancer is the third most common cancer in terms of incidence and second in terms of mortality^1^. Incidence rates were more than three times higher in countries that completed the socioeconomic transition with a very high/high HDI (Human Development Index) than in transitioning countries with a medium/low HDI. In contrast, mortality rates were more than twice as high. The highest incidence rates were reported in Europe, Australia/New Zealand, and Northern America, in Denmark in men and Norway in women. Despite recent declining or stabilizing trends for all age groups combined, several reports have documented an increase in the incidence of disease among people under 50 in some high-income countries.

Several risk factors cause the development and progression of colorectal cancer, and its incidence can be reduced by proper diet and lifestyle^2^.

Surgery is the primary treatment, and chemotherapy and/or radiotherapy may be used before or after surgery^2,3^.

Some natural compounds can sensitize to cytotoxic therapy by enhancing the effective chemotherapeutic agent concentration, the combined action of two therapeutics, or cytotoxic action specifically against cancer cells^3–8^. Such combination therapy affects multiple signaling pathways and relies on different mechanisms to reduce the development of drug resistance.

*In vitro* and *in vivo* studies have reported the effects of various land snail tissues against colorectal cancer^2^. We confirmed that water extracts of mucus and foot tissues of popular edible farmed snails *Helix aspersa aspersa* reduced the viability of human colorectal adenocarcinoma Caco-2 cells^9^. *H. a. aspersa* mucus and subunits of hemocyanins, hemolymph glycoproteins decreased the viability of human HT-29 colorectal adenocarcinoma cells^10^. These cells’ IC50 (half maximal inhibitory concentrations) were smaller than for fibroblasts. Induction of apoptosis by these compounds was also observed. In turn, Atta et al. (2021) showed that mucin derived from *Eremina desertorum* increased the expression levels of tumor suppressor genes cellular tumor antigen p53, Rb (retinoblastoma protein), APC (adenomatous polyposis coli) and PTEN (phosphatase and tensin homolog) in Caco-2 cells^11^. It also enhanced the expression levels of antioxidant marker genes GSTA1 (glutathione S-transferase alpha 1), catalase, SOD (superoxide dismutase), and GPx (glutathione peroxidase) in these cells. In another study, immunization with *Helix pomatia* hemocyanins inhibited tumor growth and lung metastasis, improved the humoral antitumor response, splenomegaly, and prolonged survival in a mouse model of colon cancer^12^. In a study by Stoyanova et al. (2020), *H. pomatia* hemocyanins supressed tumor growth, stimulated the immune system, and prolonged life span in a mouse model of colon cancer^13^. In our last article, we showed that the water extracts from *H. a. maxima* eggs (25, 25 × 10^−1^ and 25 × 10^−4^ mg/mL) and *H. a. aspersa* eggs (25, 25 × 10^−1^ and 25 × 10^−3^ mg/mL) decreased the viability of Caco-2 cells after 24 h of treatment^2^. Cell viability was reduced by fractions of extracts containing particles of molecular weight < 3 kDa (1.25 and 0.125 mg/mL). In addition, the extracts from *H. a. maxima* eggs (25, 25 × 10^−1^ and 25 × 10^−3^ mg/mL) and *H. a. aspersa* eggs (25 × 10^−1^ mg/mL) decreased the integrity of Caco-2 cell membranes. Moreover, the egg extracts (2.5 mg/mL) tended to increase the amount of lipid peroxidation products in these cells. The extracts (25 mg/mL) also caused the induction of apoptosis and reduction of necrosis. The anticancer effects may be related to the content of antioxidants, phenols, lipid peroxidation products, some proteins and peptides, methionine restriction, proper EAA (essential amino acids)/NEAA (non-essential amino acids) ratio, the content of vitamin D_3_, Ca, Mg, S, Cu, Mn, Zn, Se, and other bioactive compounds and their interactions.

The favorable chemical composition of snails has led many companies in recent years to apply for the registration of products derived from them as novel foods and human dietary supplements. *H. a. maxima* mucus is being tested for safety by EFSA (the European Food Safety Authority) as a novel food in the European Union, a dietary supplement in liquid form for adults except for pregnant women^14^. In Poland, registration is being sought for dietary supplements containing snail products, such as *H. aspersa* mucus and *H. pomatia* extract, in syrup and powder form, respectively^15^.

On the other hand, understanding the body’s physiological response to a given food requires understanding the complex digestive processes in the gastrointestinal tract^16^. For this purpose, *in vitro* models simulating digestion have been created for decades. One physiologically relevant model is the static digestion method, in which a constant ratio of food to digestive fluids and pH are used at each stage of digestion, i.e., oral, gastric, and intestinal. This method uses factors such as dilution, electrolytes, bile, enzymes, pH, and time based on physiological data. It is used to study the release of nutrients from a food matrix and to analyze the digestion products.

Two studies were conducted on hydrolyzates from *H. a. maxima* tissues, obtained using the industrial enzyme protease Alcalase®^17,18^. Hydrolyzate from hepatopancreas had the angiotensin I converting enzyme (ACE) inhibitory activity *in vitro.* It maintained this activity at a similar level after simulated gastrointestinal digestion, even exceeding the activity for non-hydrolyzed sample undergoing gastrointestinal digestion^17^. It had a considerable concentration of seven dipeptides - ACE inhibitors. In low concentrations, it also increased the metabolic activity of the Caco-2 cell monolayer as a model of the small intestinal epithelium. Moreover, the safety of the hydrolyzate in the acute toxicity study in rats was confirmed, lowering the systolic blood pressure of the spontaneously hypertensive rat model. Hydrolyzate from *H. a. maxima* snails was also characterized by significant inhibitory activity of the ACE *in vitro*, and several peptides present in it contained amino acid sequences identified in a previous study as ACE inhibitors^17,18^. In other studies, it was shown that hydrolyzates from wheat bran, additionally subjected to simulated static digestion, decreased colony formation of HCT-116 and HT-29 colon cancer cells; for the latter cells, this effect was obtained only after digestion^16,19^. In the research of Vilcacundo et al. (2018), hydrolyzate of quinoa protein concentrate, obtained by static digestion method, reduced the viability of Caco-2 colon cancer cells, indicating the release of anticancer peptides after hydrolysis^20^. Peptides from the > 5 kDa fraction showed a stronger effect than those from the < 5 kDa fraction. In turn, hydrolyzate from *Phaseolus vulgaris* also diminished the viability of colon cancer cells, HT-29 and HCT-116 lines, reduced HT-29 colony formation and caused autophagy in these cells^21^.

Considering the above, potential anticancer compounds obtained after the digestion of snail eggs may hurt the growth and development of colon cancer cells, as evaluated on Caco-2 human epithelial colorectal adenocarcinoma cells.

The research aimed to prepare hydrolyzates from lyophilized eggs of the edible snails *Helix aspersa maxima* and *Helix aspersa aspersa* as potentially bioavailable fractions. Then, selected redox status indicators, total protein and total carbohydrate content, electrophoretic profile of proteins and glycoproteins, as well as the concentration of uronic acids, allantoin, and glycolic acid in the hydrolyzates were compared with non-digested extracts. The effect of hydrolyzates on the integrity of the plasma membrane of Caco-2 cells and the monolayer of IEC-6 rat intestinal epithelial cells was determined. Then, the influence of hydrolyzates on the production of reactive oxygen species (ROS) by Caco-2 cells, the potential of their mitochondrial membrane, and the production of apoptotic proteins were examined.

## Results

### Redox status indicators

**Fig. 1:**
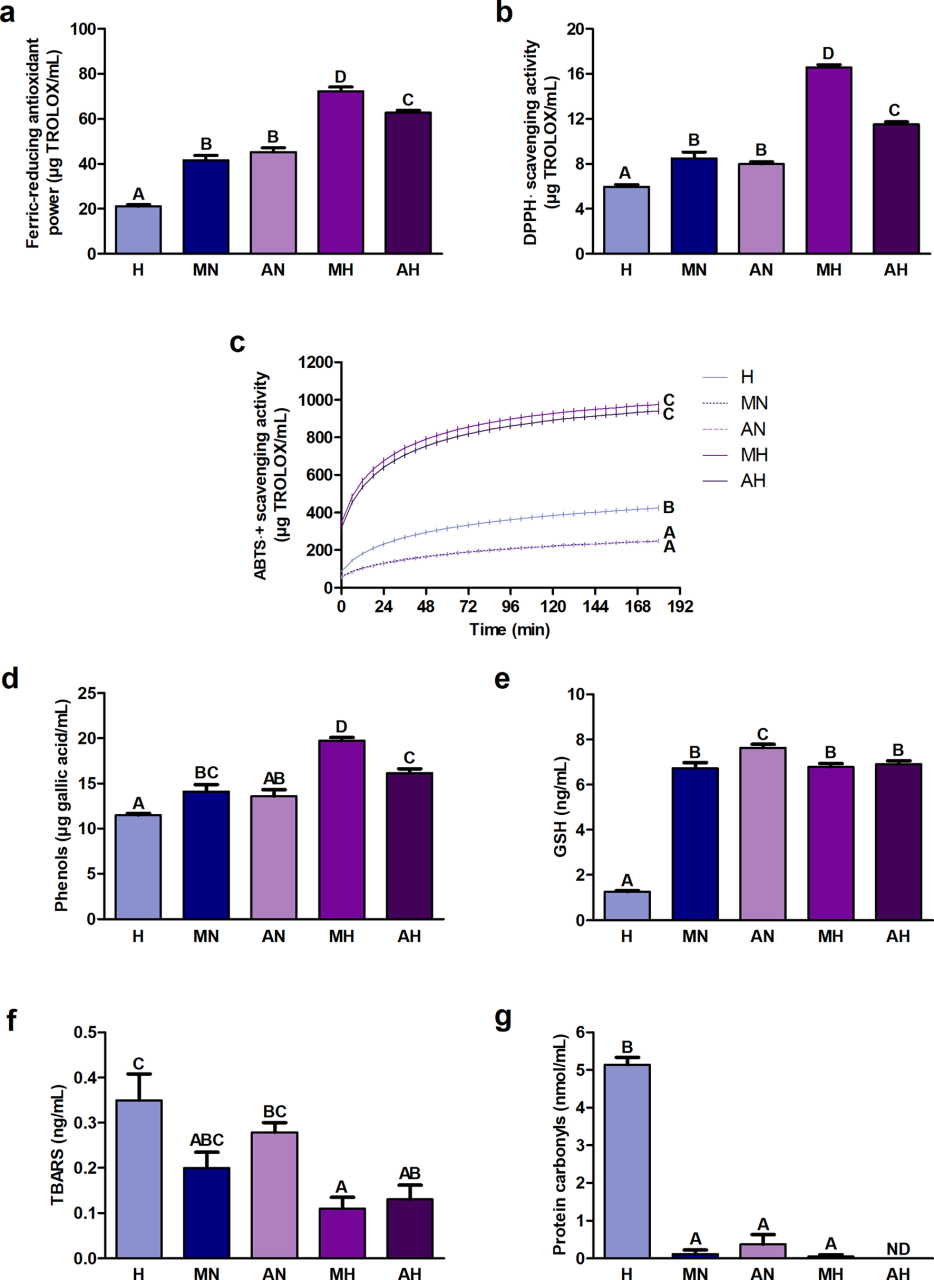
Redox status indicators: (**a**) Ferric-reducing antioxidant power; (**b**) DPPH· (2.2-diphenyl-1-picrylhydrazyl radical) scavenging activity; (**c**) ABTS·^+^ ((2.2′-azino-bis(3-ethylbenzothiazoline-6-sulfonic acid) radical cation)) scavenging activity kinetics; content of (**d**) phenols; (**e**) GSH (glutathione); (**f**) TBARS (thiobarbituric acid reactive substances) and (**g**) protein carbonyls in hydrolyzates from *Helix aspersa maxima* and *Helix aspersa aspersa* eggs. H, digestive fluids; MN and AN, non-digested extracts from *H. a. maxima* and *H. a. aspersa* eggs, respectively; MH and AH, hydrolyzates from *H. a. maxima* and *H. a. aspersa* eggs, respectively; ND, not detected. Error bars show the standard error of the mean. Values of one indicator without a common letter (A-D) differ statistically significantly (*p* < 0.01). *n* = 6 (all analyzes except protein carbonyls), *n* = 3 (protein carbonyls).

The ferric-reducing antioxidant power and DPPH· (2.2-diphenyl-1-picrylhydrazyl radical) scavenging activity were significantly higher in hydrolyzates than in non-digested extracts (Fig. 1a, b). The highest values were recorded in the hydrolyzate from *H. a. maxima* eggs and the lowest - in digestive fluids. ABTS·^+^ ((2.2′-azino-bis(3-ethylbenzothiazoline-6-sulfonic acid) radical cation)) scavenging activity was significantly higher in hydrolyzates compared to non-digested extracts (Fig. 1c). Its value in non-digested extracts was significantly lower than in digestive fluids. The content of phenols was significantly greater in hydrolyzates than in non-digested extracts and digestive fluids (Fig. 1d). The hydrolyzate from *H. a. maxima* eggs had the highest concentration. The content of glutathione (GSH) in the hydrolyzate from *H. a. maxima* eggs did not differ significantly from its content in the hydrolyzate from *H. a. aspersa* eggs and in the non-digested extract from *H. a. maxima* eggs (Fig. 1e). The GSH level in the hydrolyzate from *H. a. aspersa* eggs was significantly lower than in the corresponding non-digested extract. Significantly the least amount of GSH was found in digestive fluids. The concentration of lipid peroxidation products, thiobarbituric acid reactive substances (TBARS), was significantly lower in hydrolyzates compared to digestive fluids, and statistically significant differences were not observed in the case of non-digested extracts (Fig. 1f). The level of protein oxidation products, protein carbonyls, was significantly lower in the hydrolyzate from *H. a. maxima* eggs and non-digested extracts than in digestive fluids (Fig. 1g). No protein carbonyls were detected in the hydrolyzate from *H. a. aspersa* eggs.

### Content of total protein and total carbohydrates

**Fig. 2:**
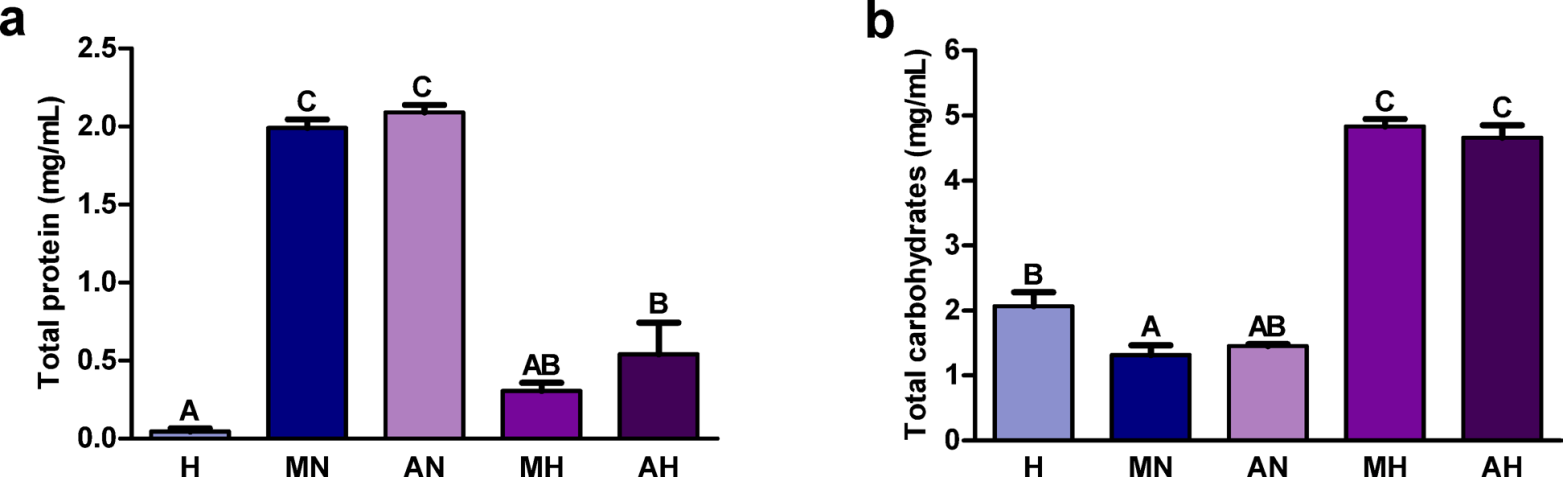
Content of (**a**) total protein and (**b**) total carbohydrates in hydrolyzates from *Helix aspersa maxima* and *Helix aspersa aspersa* eggs. H, digestive fluids; MN and AN, non-digested extracts from *H. a. maxima* and *H. a. aspersa* eggs, respectively; MH and AH, hydrolyzates from *H. a. maxima* and *H. a. aspersa* eggs, respectively. Error bars show the standard error of the mean. Values of one indicator without a common letter (A-C) differ statistically significantly (*p* < 0.01). *n* = 6 (total protein), *n* = 3 (total carbohydrates).

The total protein level was significantly lower in the hydrolyzates than in the corresponding non-digested extracts (Fig. 2a). The protein content in the hydrolyzate from *H. a. maxima* eggs did not differ significantly from its content in digestive fluids. The concentration of total carbohydrates was significantly higher in hydrolyzates than in non-digested extracts and digestive fluids (Fig. 2b).

### SDS-PAGE (sodium dodecyl sulfate-polyacrylamide gel electrophoresis) profile of proteins and glycoproteins

**Fig. 3:**
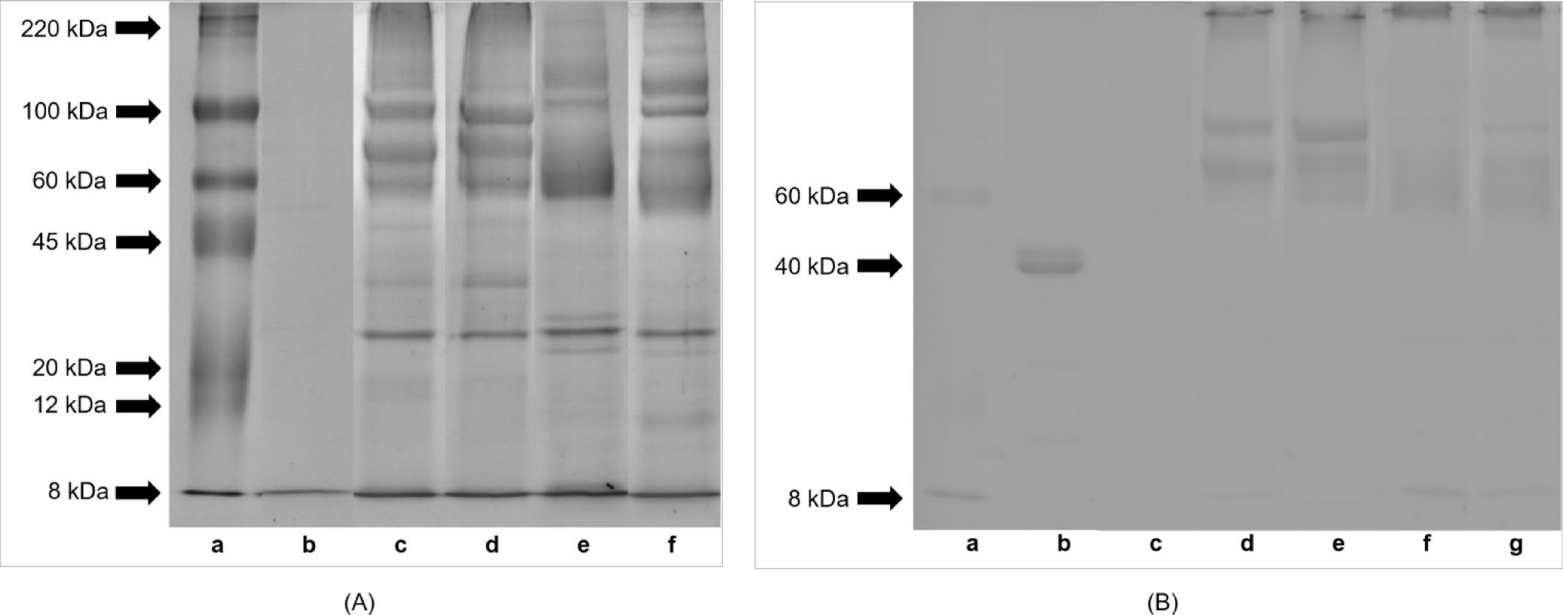
(**A**) Protein and (**B**) glycoprotein profile of hydrolyzates from *Helix aspersa maxima* and *Helix aspersa aspersa* eggs, obtained by SDS-PAGE. (**A**) (a) protein molecular weight marker (Sigma-Aldrich, St. Louis, MO, USA); (b) digestive fluids; (c) non-digested extract from *H. a. maxima* eggs; (d) non-digested extract from *H. a. aspersa* eggs; (e) hydrolyzate from *H. a. maxima* eggs and (f) hydrolyzate from *H. a. aspersa* eggs. (**B**) (a) protein molecular weight marker (Sigma-Aldrich, St. Louis, MO, USA); (b) horseradish peroxidase (positive control); (c) digestive fluids; (d) non-digested extract from *H. a. maxima* eggs; (e) non-digested extract from *H. a. aspersa* eggs; (f) hydrolyzate from *H. a. maxima* eggs and (g) hydrolyzate from *H. a. aspersa* eggs. Original gels are presented in Supplementary Figure S1.

The hydrolyzates and non-digested extracts contained proteins and peptides in the molecular weight range of standard proteins, i.e., 8–220 kDa (Fig. 3A, panel c-f, Supplementary Table S1). Proteins larger than approximately 50 kDa predominated (Fig. 3A, panel c-f, Table 1, Supplementary Table S1). Hydrolyzates contained more proteins and peptides smaller than approximately 20 kDa and fewer proteins larger than approximately 20 kDa compared to non-digested extracts.

The presence of glycoproteins with a molecular weight of 8–220 kDa was detected in hydrolyzates and non-digested extracts (Fig. 3B, panel d-g, Supplementary Table S1). Glycoproteins larger than approximately 50 kDa predominated, especially those with a molecular weight of approximately 50–100 kDa (Fig. 3B, panel d-g, Table 1, Supplementary Table S1). Hydrolyzates contained fewer glycoproteins from this range than non-digested extracts, and the band corresponding to 8 kDa is more pronounced.

**Table 1:** Densitometric analysis of protein and glycoprotein profile of hydrolyzates from *Helix aspersa maxima* and *Helix aspersa aspersa* eggs, obtained by SDS-PAGE (shown in Fig. 3). Integrated density measurements are presented in Supplementary Table S1.

Concentration (%)	*H. a. maxima* non-digested extract	*H. a. aspersa* non-digested extract	*H. a. maxima* hydrolyzate	*H. a. aspersa* hydrolyzate
Proteins > 50 kDa	57	60	55	60
Proteins < 20 kDa in the hydrolyzate relative to its non-digested extract	-	-	112	145
Proteins > 20 kDa in the hydrolyzate relative to its non-digested extract	-	-	98	99
Glycoproteins > 50 kDa	77	79	61	68
Glycoproteins 50–100 kDa in > 50 kDa fraction	76	70	68	71
Glycoproteins 50–100 kDa in the hydrolyzate relative to its non-digested extract	-	-	45	64
Glycoproteins ~ 8 kDa in the hydrolyzate relative to its non-digested extract	-	**-**	202	154

### Content of uronic acids, allantoin, and glycolic acid

**Fig. 4:**
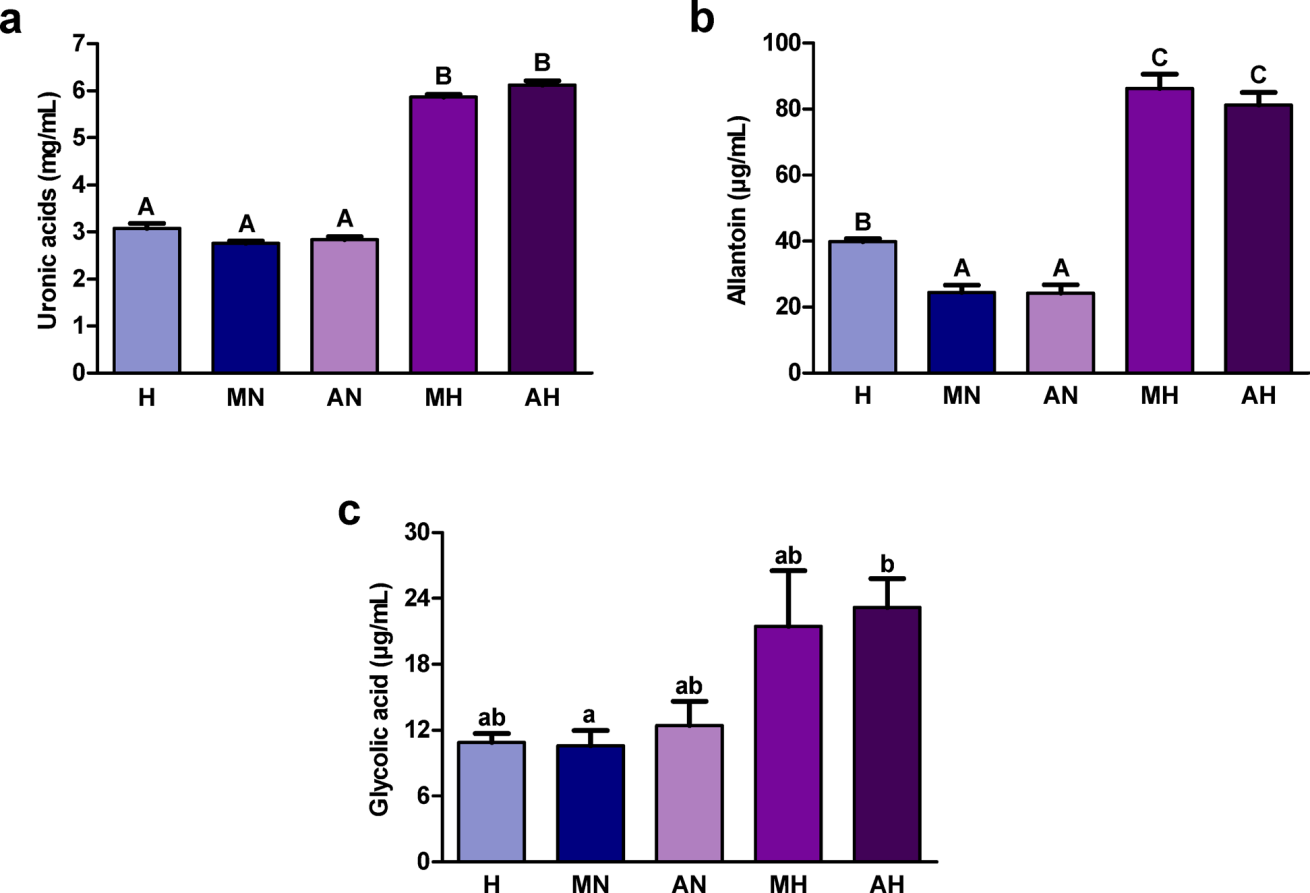
Concentration of (**a**) uronic acids; (**b**) allantoin; and (**c**) glycolic acid in hydrolyzates from *Helix aspersa maxima* and *Helix aspersa aspersa* eggs. H, digestive fluids; MN and AN, non-digested extracts from *H. a. maxima* and *H. a. aspersa* eggs, respectively; MH and AH, hydrolyzates from *H. a. maxima* and *H. a. aspersa* eggs, respectively. Error bars show the standard error of the mean. Values of one indicator without a common letter differ statistically significantly (a, b – at *p* < 0.05; A-C – at *p* < 0.01). *n* = 6 (uronic acids), *n* = 5 (allantoin), *n* = 4 (glycolic acid).

The content of uronic acids was significantly higher in hydrolyzates than in non-digested extracts and digestive fluids (Fig. 4a). The level of allantoin was significantly greater in hydrolyzates compared to non-digested extracts, in which the concentration of this compound was significantly lower than in digestive fluids (Fig. 4b). We observed a trend towards higher glycolic acid content in hydrolyzates compared to non-digested extracts and digestive fluids (Fig. 4c).

### Effect of hydrolyzates on the integrity of plasma membranes of Caco-2 and IEC-6 cells

**Fig. 5:**
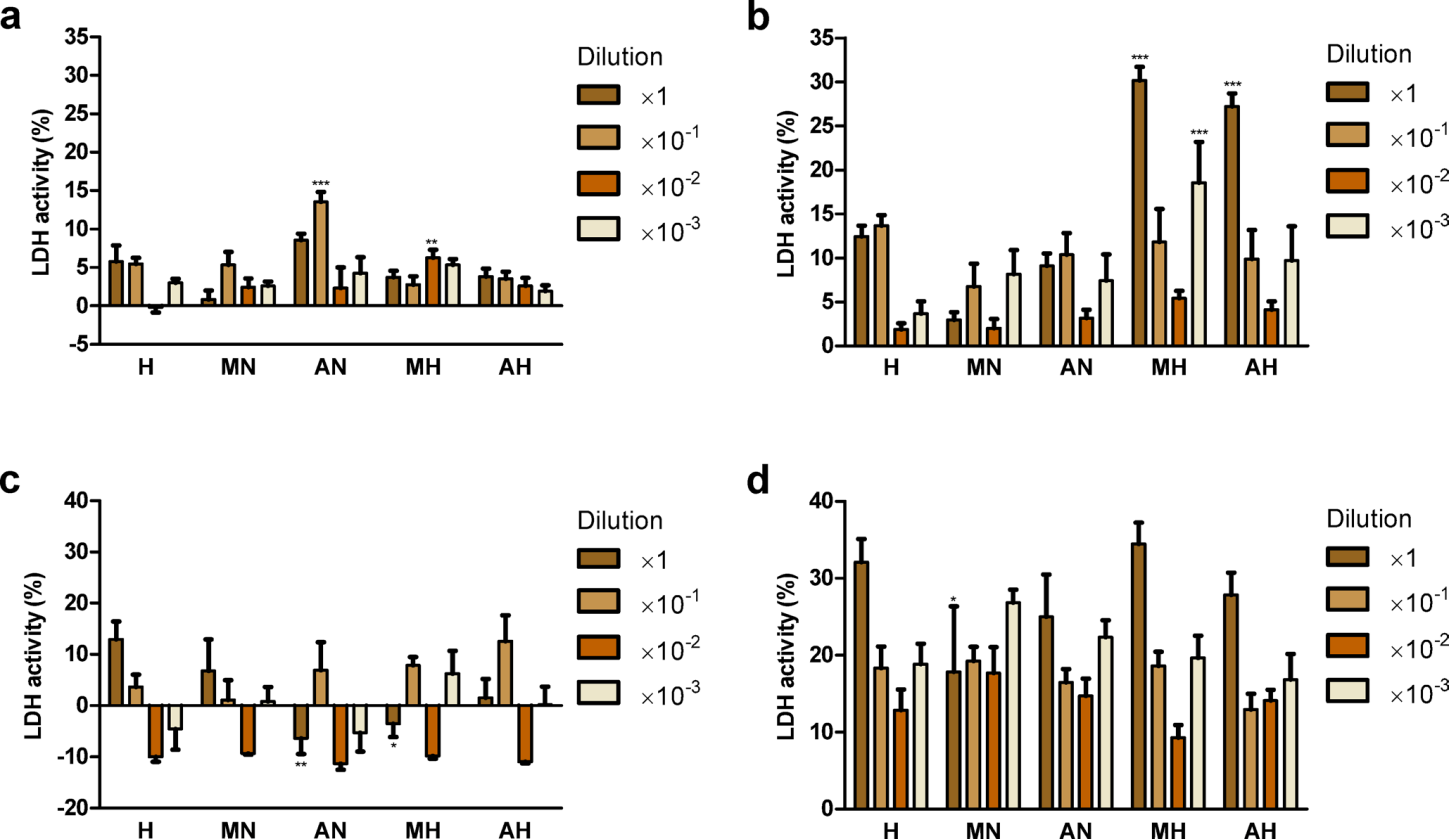
The integrity of the plasma membrane of Caco-2 cells incubated with hydrolyzates from *Helix aspersa maxima* and *Helix aspersa aspersa* eggs (concentrated and diluted 10, 100, and 1000 times) for (**a**) 24 h and (**b**) 72 h; and integrity of plasma membrane of IEC-6 cells treated with these hydrolyzates for (**c**) 24 h and (**d**) 72 h. H, digestive fluids; MN and AN, non-digested extracts from *H. a. maxima* and *H. a. aspersa* eggs, respectively; MH and AH, hydrolyzates from *H. a. maxima* and *H. a. aspersa* eggs, respectively; LDH, lactate dehydrogenase. Error bars show the standard error of the mean. An asterisk (*) indicates values that differ from control (for cells treated with H of appropriate concentration) at *p* < 0.05, two asterisks (**) - at *p* < 0.01, and three asterisks (***) – at *p* < 0.001. *n* = 6.

Treatment with hydrolyzate from *H. a. maxima* eggs and its dilutions (10^−1^, 10^−3^) and hydrolyzate from *H. a. aspersa* eggs and its dilutions (10^−1^, 10^−2^, 10^−3^), for 24 h, did not significantly affect the integrity of the plasma membrane of Caco-2 cells, the concentration of lactate dehydrogenase (LDH) released from the cells, compared to control cells, incubated with digestive fluids at the corresponding concentration (Fig. 5a). The exception was *H. a. maxima* egg hydrolyzate (10^−2^ dilution), which increased LDH release. Incubation with concentrated hydrolyzates and with hydrolyzate from *H. a. maxima* eggs (10^−3^ dilution) for 72 h significantly increased the release of this enzyme (Fig. 5b). Treatment with concentrated *H. a. maxima* egg hydrolyzate for 24 h significantly reduced LDH release from IEC-6 cells (Fig. 5c). Incubation with hydrolyzates for 72 h had no significant effect on the integrity of the plasma membrane of these cells (Fig. 5d). The negative values in Fig. 5a and c are due to the inclusion of results for additional control cells treated with deionized water (spontaneous LDH activity) in the calculations.

### Influence of hydrolyzates on the content of ROS in Caco-2 cells

**Fig. 6:**
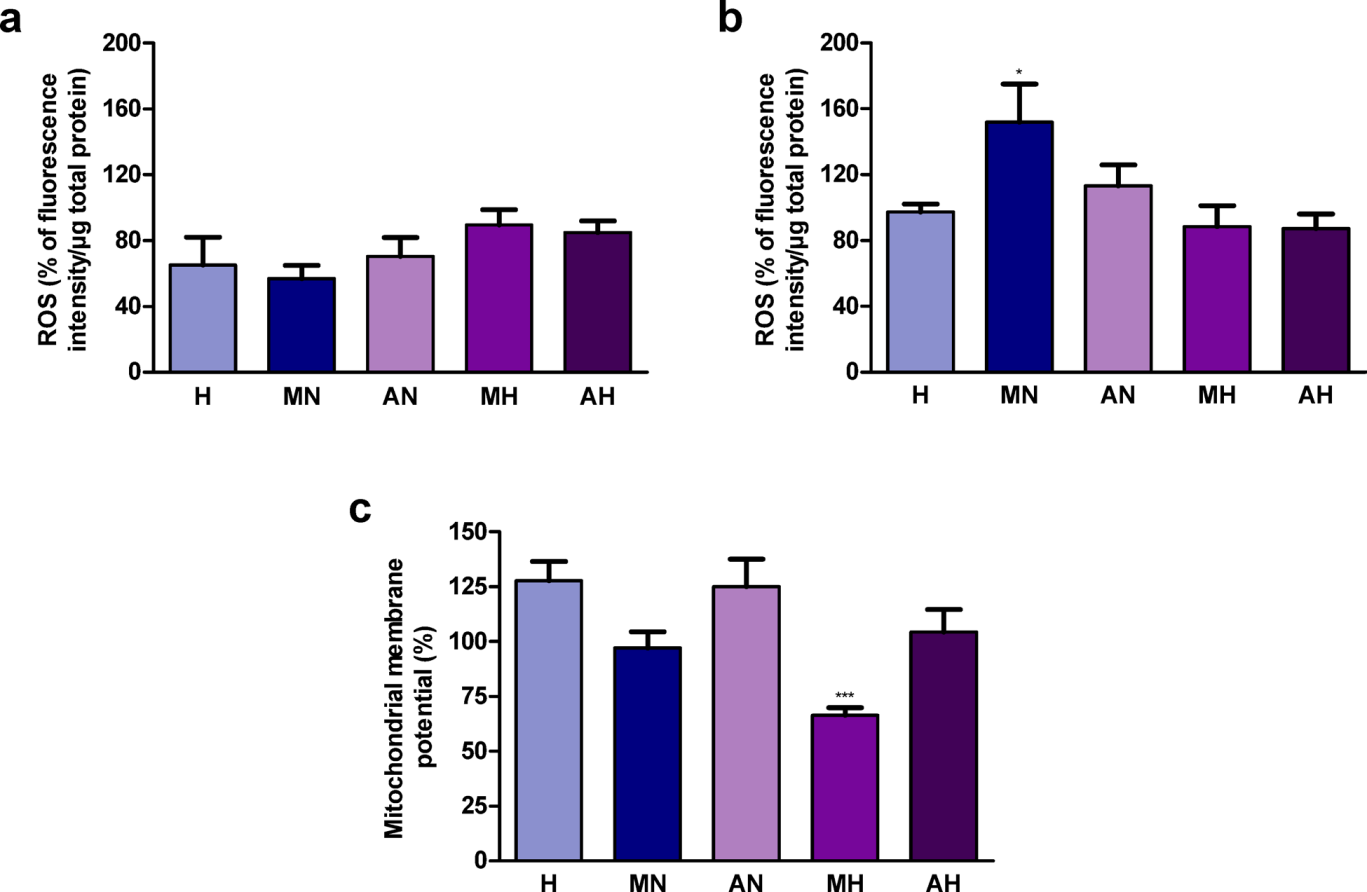
Content of ROS (reactive oxygen species) in Caco-2 cells treated with hydrolyzates from *Helix aspersa maxima* and *Helix aspersa aspersa* eggs for (**a**) 24 h and (**b**) 72 h and (**c**) mitochondrial membrane potential of these cells incubated with hydrolyzates for 72 h. H, digestive fluids; MN and AN, non-digested extracts from *H. a. maxima* and *H. a. aspersa* eggs, respectively; MH and AH, hydrolyzates from *H. a. maxima* and *H. a. aspersa* eggs, respectively. The results are expressed as % of the value of control cells incubated with deionized water. Error bars show the standard error of the mean. An asterisk (*) indicates a value that differs from the control (for cells treated with H) at *p* < 0.05 and three asterisks (***) – at *p* < 0.001. *n* = 6 (ROS), *n* = 5 (mitochondrial membrane potential).

Treatment of Caco-2 cells with hydrolyzates for 24 and 72 h did not affect their ROS content (Fig. 6a, b).

### Effect of hydrolyzates on the mitochondrial membrane potential of Caco-2 cells

Incubation with the hydrolyzate from *H. a. maxima* eggs for 72 h significantly reduced the mitochondrial membrane potential of Caco-2 cells; in the case of the hydrolyzate from *H. a. aspersa* eggs, a tendency to reduce the mitochondrial membrane potential of these cells was observed (Fig. 6c).

### Influence of hydrolyzates on the production of apoptotic proteins by Caco-2 cells

Treatment of Caco-2 cells for 72 h with *H. a. maxima* egg hydrolyzate significantly increased the production of the following apoptotic proteins: apoptosis regulator Bcl-W (Bcl-W), BID, Bcl-2-like protein 11 (BIM), and death receptor 6 (DR6) (Table 2). Incubating these cells with *H. a. aspersa* egg hydrolyzate significantly increased the apoptosis regulator Bcl-W, BID, TRAIL-R2 (TNF-related apoptosis-inducing ligand receptor 2), and TRAIL-R3 production. There was also a tendency for this hydrolyzate to increase SMAC (second mitochondria-derived activator of caspases) protein concentration.

**Table 2:** The concentration of apoptotic proteins produced by Caco-2 cells during 72 h incubation with hydrolyzates from *Helix aspersa maxima* and *Helix aspersa aspersa* eggs.

Protein (%)	Digestive fluids	*H. a. maxima* hydrolyzate	*H. a. aspersa* hydrolyzate	*p*
BAD	103.87 ± 7.26	113.46 ± 4.11	104.37 ± 4.93	0.485
Apoptosis regulator BAX	104.70 ± 1.66	101.11 ± 2.31	104.02 ± 1.05	0.417
Apoptosis regulator Bcl-2	103.88 ± 0.55	102.40 ± 2.67	107.29 ± 1.88	0.317
**Apoptosis regulator Bcl-W**	96.51 ± 0.33^A^	**111.12 ± 0.95** ^**B**^	**106.60 ± 2.04** ^**B**^	0.009
**BID**	87.85 ± 3.27^A^	**123.11 ± 1.24** ^**C**^	**104.65 ± 2.26** ^**B**^	0.004
**Bcl-2-like protein 11**	93.23 ± 0.27^a^	**109.04 ± 1.91** ^**b**^	101.82 ± 3.73^ab^	0.043
Caspase-3	ND	96.03 ± 0.75	79.91 ± 10.37	0.261
Caspase-8	104.40 ± 2.17	104.99 ± 0.60	114.11 ± 4.14	0.142
CD40	97.76 ± 0.88	95.33 ± 1.41	99,87 ± 0.28	0.100
CD40 ligand	103.45 ± 2.66	99.33 ± 3.91	104,81 ± 0.88	0.449
C-IAP2	93.21 ± 4.51	96.59 ± 5.24	103.23 ± 2.99	0.377
Cytochrome c	110.14 ± 1.32	105.58 ± 2.30	102.74 ± 0.23	0.091
**Death receptor 6**	117.58 ± 1.60^A^	**136.52 ± 0.34** ^**B**^	112.65 ± 2.74^A^	0.005
Apoptosis-mediating surface antigen FAS	99.91 ± 0.67	100.49 ± 0.88	94.02 ± 2.39	0.098
FasL	101.37 ± 0.85	104.41 ± 3.85	100.08 ± 0.97	0.494
HSP 27	103.68 ± 1.75	109.28 ± 3.32	105.55 ± 2.13	0.389
HSP 60	108.15 ± 1.51	110.99 ± 3.90	109.44 ± 3.59	0.829
HSP 70	105.23 ± 3.08	107.32 ± 0.01	103.75 ± 0.91	0.482
Serine protease HTRA1	97.36 ± 1.95	101.27 ± 2.44	101.53 ± 1.04	0.349
IGF-I	89.85 ± 5.97	108.47 ± 5.22	106.72 ± 5.22	0.163
IGF-II	105.93 ± 2.01	109.91 ± 0.91	112.57 ± 8.27	0.671
IGF-I soluble receptor	84.81 ± 2.37	86.04 ± 1.04	92.63 ± 0.98	0.075
IGFBP-1	71.85 ± 8.59	82.39 ± 1.26	90.97 ± 6.17	0.236
IGFBP-2	65.23 ± 10.64	74.87 ± 1.98	90.55 ± 3.34	0.150
IGFBP-3	92.88 ± 4.10	89.05 ± 2.32	98.45 ± 1.57	0.211
IGFBP-4	104.61 ± 5.54	99.14 ± 1.57	113.27 ± 1.26	0.129
IGFBP-5	105.04 ± 2.99	99.95 ± 4.02	110.02 ± 0.94	0.197
IGFBP-6	93.48 ± 1.11	93.22 ± 2.60	97.35 ± 0.70	0.293
Livin	84.26 ± 2.34	88.29 ± 3.90	90.02 ± 1.37	0.423
p21	97.23 ± 2.40	98.64 ± 3.40	105.59 ± 0.11	0.165
Cyclin-dependent kinase inhibitor p27	90.71 ± 3.99	92.43 ± 3.23	101.18 ± 0.61	0.162
Cellular tumor antigen p53	83.47 ± 3.95	89.12 ± 5.81	98.31 ± 0.82	0.171
**SMAC**	84.30 ± 1.82^a^	87.88 ± 3.21^ab^	**97.33 ± 0.82** ^**b**^	0.050
sTNF-R1	101.54 ± 3.44	103.22 ± 5.17	117.18 ± 1.61	0.102
sTNF-R2	57.93 ± 4.72	76.46 ± 0.36	82.91 ± 9.60	0.128
Apoptosis inhibitor survivin	98.75 ± 0.37	95.28 ± 4.23	108.05 ± 1.06	0.076
TNF-alpha	78.10 ± 6.82	80.67 ± 5.43	98.61 ± 3.47	0.133
TNF-beta	87.07 ± 3.08	81.86 ± 0.83	96.55 ± 4.22	0.090
TRAIL-R1	82.72 ± 5.29	76.90 ± 1.53	96.86 ± 2.15	0.054
**TRAIL-R2**	99.09 ± 3.11^a^	93.82 ± 2.89^a^	**119.32 ± 1.27** ^**b**^	0.012
**TRAIL-R3**	72.55 ± 4.16^a^	65.67 ± 1.21^a^	**90.20 ± 1.00** ^**b**^	0.014
TRAIL-R4	68.75 ± 5.26	74.27 ± 4.23	88.19 ± 0.98	0.082
E3 ubiquitin-protein ligase XIAP	97.21 ± 3.12^ab^	93.69 ± 0.08^a^	110.00 ± 3.37^b^	0.044

Note: Control cells were treated with digestive fluids. The results are expressed as % of the content in second control cells incubated with deionized water as mean ± standard error of the mean. Values for a given protein without a common superscript differ statistically significantly (a, b – at *p* < 0.05; A-C - at *p* < 0.01). *n* = 2.

## Discussion

High cell content of reactive oxygen species (ROS) could result in oxidative stress, and antioxidants prevent their formation and inhibit their activity^2^. Certain antioxidants could have beneficial effects on colorectal cancer initiation and progression. They could reduce cell proliferation, activate apoptosis, and reduce metastasis and resistance to chemotherapeutics. In our study, greater general antioxidant activity was recorded in hydrolyzates than in non-digested extracts, and the ferric-reducing antioxidant power and DPPH· scavenging activity of *H. a. maxima* hydrolyzate were greater than that of *H. a. aspersa*. Differences in the antioxidant activity of hydrolyzates determined by different methods are mainly due to the different mechanisms of these methods^2^. DPPH· scavenging activity involves measuring the ability of antioxidants to donate electrons, like the other two methods, and on the transfer of hydrogen atom. On the other hand, in the case of the ferric-reducing antioxidant power, Fe^3+^ reduction is monitored. Moreover, DPPH· is less reactive than ABTS·^+^ and reflects hydrophilic and high-pigmented compounds less well. The general antioxidant indices considered together indicate the release of antioxidants during hydrolysis.

The increased antioxidant potential of hydrolyzates relative to non-digested extracts was most likely mainly due to the presence of low molecular weight antioxidant peptides in them, especially < 3 kDa^22,23^ and we confirmed the higher content of peptides smaller than 12 kDa by SDS-PAGE. The antioxidant action of peptides could result from the scavenging of ROS and the chelating of transition metal ions^24^. Some amino acids’ content, location, and configuration of the peptide affect its interaction with free radicals. Peptides provide hydrogen, which reacts with free radicals to create stable products^22^. Hydrophobic amino acids contained in them, such as phenylalanine (Phe), alanine, and proline (Pro), could be responsible for the high free radical scavenging activity. Antioxidant activity could also be supported by other hydrophobic amino acids present at the N-terminus of peptides, such as leucine (Leu) or valine. Leu because of a long aliphatic side chain group that easily interacts with acyl chains found in fatty acids. Moreover, the aromatic residues of the tryptophan (Trp) and tyrosine (Tyr) could induce radicals that are deficient in electrons and provide protons in the case of interaction involving phenolic and indole groups. Histidine (His), due to the imidazole ring, can deliver protons; it chelates and traps lipid radicals. In our study, pepsin, used to create hydrolyzates, is highly specific to the residues of aromatic amino acids (Phe, Trp, Tyr, Pro), while the trypsin - to the residues of basic amino acids ((lysine (Lys), arginine (Arg), His))^24^. The specificity of proteolytic enzymes used to obtain hydrolyzates determined the size and sequence of peptides obtained, which determined their antioxidant activity. In the study of He et al. (2019), protein hydrolyzate of false abalone (*Volutharpa ampullacea perryi*), made with the use of trypsin, was characterized by a greater degree of hydrolysis and reduction of ABTS·^+^ than hydrolyzates created using pepsin and several commercial proteases^24^. The highest ability to scavenge ABTS·^+^ was demonstrated by fractions with low molecular weight, especially < 3 kDa, in which antioxidant peptides were identified. Moreover, the digestion products of compounds containing uronic acids, such as chondroitin sulfate, may also have contributed to the greater antioxidant potential of hydrolyzates relative to non-digested extracts^25^. The release of other compounds and the increase in the pH of the environment during digestion, leading to the hydroxyl group deprotonation of phenolic aromatic rings, could also have been responsible for their higher antioxidant potential^19^. In the study of Tomé-Sánchez et al. (2023), antioxidant activity, measured by methods using single electron transfer, hydrogen atom transfer, mixed mechanism, and a method using a metal ion chelating mechanism, of wheat bran hydrolyzates additionally subjected to the static digestion method increased compared to non-digested hydrolyzates^19^.

In our research, we also showed a higher content of phenols in hydrolyzates than in non-digested extracts, and their greatest content was characterized by hydrolyzate from *H. a. maxima* eggs. The results indicate the release of phenols in the hydrolysis. In turn, studies by many authors indicate the release of certain phenolic acids or related compounds during gastrointestinal digestion, for example, p-coumaric acid, ferulic acid, caffeic acid, coumaroylquinic acid 3, protocatechuic acid, sinapic acid, 5-caffeoylquinic acid, quercetin-3-glucuronide, hydroxytyrosol, tyrosol, hydroxytyrosol acetate^26–30^. According to Tomé-Sánchez et al. (2023) and Cilla et al. (2022), the bioaccessibility and stability of phenolic compounds in the gastrointestinal tract can vary depending on the food matrix, compound structure, processing, and conditions of digestion^19,31^. Phenolic compounds can bind to components such as resistant proteins, which reduces their bioaccessibility in digestive juices^19^. Moreover, mild alkaline conditions of the intestinal environment contribute to a decrease in the stability of phenols, especially multi-ring^31^. It is worth noting that these compounds, particularly low-molecular-weight, although they belong to antioxidants, may, under certain conditions, self-oxidize and behave like prooxidants^2^. However, we did not observe a pro-oxidant effect of the hydrolyzates against Caco-2 cells, in which the concentration of ROS was not increased by incubation with them. Phenols could have had cytotoxic effects on cancer cells according to other mechanisms, including apoptosis induction and cell cycle and proliferation inhibition^7^. They can modulate multiple biological pathways associated with cancer progression and treatment resistance. In addition, they act synergistically with commonly used chemotherapeutics and natural compounds.

GSH is a main intracellular antioxidant that regulates the redox state of cells, controlling many cellular processes, including proliferation and apoptosis^2^. Disturbances in its homeostasis are associated with tumor initiation, progression, and effectiveness of treatment. In our study, the concentration of GSH in the hydrolyzates from the eggs of both snail subspecies was similar, but in the case of the hydrolyzate from the eggs of *H. a. aspersa* there was a decrease in its content relative to the non-digested extract, indicating that some amount of this compound was digested.

Vassilev et al. (2020) detected the presence of similar antioxidant metabolites in two fractions, with particle sizes < 1 kDa and < 3 kDa, of *H. a. aspersa* mucus^32^.

The concentration of lipid peroxidation products in the hydrolyzates was significantly lower compared to the digestive fluids, and no significant differences were observed in the case of the non-digested extracts. Moreover, the concentration of metal-catalyzed oxidation products, protein carbonyls, was significantly lower in *H. a. maxima* egg hydrolyzate and non-digested extracts than in digestive fluids. Their content in the hydrolyzate from *H. a. maxima* eggs was very low, and in the hydrolyzate from *H. a. aspersa* eggs, they were not detected at all. The pro-oxidant compounds could be mainly formed during hydrolysis, and additional antioxidants released and formed during this process could effectively reduce oxidation, especially of proteins. Other authors demonstrated that malondialdehyde (MDA), a lipid peroxidation product, reacts with nucleic acid bases to form adducts, leading to apoptosis^2^. It was also confirmed that another lipid peroxidation product, HNE (4-hydroxynonenal) can induce apoptosis by affecting oxidative homeostasis, which we did not observe on Caco-2 cells incubated with snail egg hydrolyzates.

Snail egg hydrolyzates’ considerably lower total protein level than non-digested extracts indicates that it was partially digested into low-molecular-weight proteins and peptides during hydrolysis. It is important to note that although the Bradford assay is widely used for protein quantification, it has limited sensitivity toward short peptides and low-molecular-weight proteins, it does not detect peptides and proteins with molecular weight lower than approximately 3–5 kDa (information from the manufacturer of the Bradford reagent, Bio-Rad Laboratories)^33,34^. Bradford reagent (Coomassie brilliant blue G-250 dye) exists in neutral, cationic, and anionic forms. Although the anionic form is not present at the pH of the Bradford reagent, it forms a complex with the protein. Binding of the dye requires a macromolecule and interactions occurring mainly with Arg rather than with primary amino groups, and to a lesser extent with other basic (Lys, His) and aromatic (Trp, tyrosine, and Phe) residues. Moreover, binding behavior is related to hydrophobic interactions and Van der Waals forces. Adequate protein content in the diet is very important for cancer prophylaxis. In a study by Ho et al. (2011), human colorectal carcinoma and mouse squamous cell carcinoma VII developed more slowly in mice receiving a high-protein, low-carbohydrate diet than in animals that consumed a low-protein, high-carbohydrate diet due to a reduction in glycolysis^35^. In contrast, in research by Rubio-Patiño et al. (2018) on mouse models of cancer, administering a low-protein diet reduced tumor growth^36^. In our research, the hydrolyzates contained proteins and peptides in the 8-220 kDa range, and proteins > 50 kDa predominated. Compared to non-digested extracts, they comprised more proteins and peptides < 20 kDa. It is noteworthy that the colloidal Coomassie G-250 stain method has an advantage in sensitivity in detecting such peptides and low-molecular-weight proteins compared to the Bradford method^34,37^. Staining on the gel is done after SDS-PAGE - each molecular fraction is separated by molecular weight and individually stained. The dye signal is concentrated exactly at the site of the protein stripe, rather than throughout the solution, thus increasing the ratio of signal to background, which is clear. The dye forms micellar aggregates, intensely staining proteins. Glycoproteins with a molecular weight of 8-220 kDa were also detected in the hydrolyzates, and glycoproteins > 50 kDa, especially 50–100 kDa, predominated. The hydrolyzates contained fewer glycoproteins in this range than the non-digested extracts and more glycoproteins < 8 kDa. These results, like the total protein concentration results, indicate that the proteins and glycoproteins of snail eggs are partially digested during hydrolysis, and low-molecular-weight proteins and peptides are formed in the process. It was shown that short peptides are well-diffusive and mobile and, therefore, interact well with components of cancer cells^22^. Moreover, hydrophobic amino acids can ameliorate the interactions between anticancer peptides and outer leaflets of cancer cell membrane bilayers. This gives them a solid cytotoxic effect, selectively directed to cancer cells. However, cellular selectivity and the degree of cell susceptibility to lysis by peptides depend on the cell membrane composition and the distribution of phospholipids. It was also proved that the anticancer properties of peptides may increase if their sequence possesses charged (glutamic acid) or heterocyclic (proline) amino acids. In the research of Prakot at al. (2018), the fraction of hydrolyzate from the defatted spotted babylon snail (*Babylonia areolata*), obtained using the alkaline serine endopeptidase Protease G6, with a molecular weight of < 3 kDa, the richest in antioxidants, reduced the viability of B16-F10 mouse melanoma cells, after a 72-h incubation, and activated apoptosis in them, after 8-h incubation^23^. In another study, chromatographic protein fractions (31–61.2 kDa) of abalone (*Haliotis fulgens*) viscera hydrolyzate, obtained using Wobenzym systemic enzyme formulation, limited the viability of grade IV prostate adenocarcinoma PC-3 cells, after 24 h of incubation, and the level of expression of the *mmp* (*matrix metalloproteinase*)−2 and *mmp*−9 genes, after 37 h of incubation, responsible for invasion and metastasis^38^. In turn, according to Gaspar-Wintiliescu et al. (2019), gelatin extracted from the sea snail *Rapana venosa*, with the help of thermal acid and enzymatic hydrolysis using pepsin, only slightly reduced the viability of HaCaT human keratinocytes, after 24 and 48 h of incubation, to a lesser extent than pig skin gelatin^39^. Moreover, sixteen putative amphipathic and cationic anticancer peptides were detected in two fractions of *Achatina fulica* mucus, which reduced MCF7 and Vero cell viability^40^. The molecular weight of the nine peptides was < 3 kDa. Anticancer peptides were also predicted in the land snail mucus^41^. In turn, the glycoprotein agglutinin contained in the *H. aspersa* snail eggs perivitelline fluid may be applied in the drug delivery systems for colorectal cancer^42^.

Then, we found a higher content of total carbohydrates in the hydrolyzates than in the non-digested extracts, which indicates their release during the hydrolysis process. According to Ansart et al. (2007) and Tompa (1976), *H. aspersa* eggshell is composed of calcium carbonate crystals bound to a mucopolysaccharide matrix, surrounded by a mucus layer^43,44^. In turn, Nicolai et al. (2012) showed that galactogen is a major compound of *H. a. aspersa* eggs, and its content in them exceeded the glycogen content by about six times^45^. Moreover, we detected the uronic acids in the hydrolyzates, also in higher concentrations than in non-digested extracts. They are a component of glycosaminoglycans (GAGs), a family of strongly acidic polysaccharides that can be divided into non-sulfated GAGs - hyaluronic acid and sulfated GAGs – in snails, mainly chondroitin sulfate and heparan sulfate^46,47^. Most polysaccharides pass into the distal colon and rectum, providing nutrition for the microbiota growth and metabolism^48^. GAGs can perform many functions in vertebrates; they are structural and organizational components of connective tissue and cell-growth factors and play a role in cell-matrix and cell-cell interactions^47^. They influence the functional properties of cells and processes such as growth, migration, and metastasis. Heparin and its polymers participate in anticoagulation and angiogenesis.

The hyaluronic acid content in *H. a. aspersa* mucus, described by Alogna et al. (2021) and Mencucci et al. (2021), was 70–80 mg/L, while the concentration of sulfated GAGs was 29–90 mg/L^46,49^. Chondroitin sulfate was found in these snails’ heart, mantle, and kidney, and heparan sulfate in smaller amounts^46^. In turn, the mucus of *Helix lucorum* and *A. fulica* contained 9.3% and 15.9% GAGs, respectively, while the body of *A. fulica* contained 1.6%^50^. The average molecular weight of these proteoglycans was 900 kDa and 696.7 kDa for *H. lucorum* and *A. fulica* mucus, respectively, and the sulfate content was 13.6% and 15.4%.

For the recognition, binding, and internalization of hyaluronic acid, a disaccharide unit of glycosaminoglycan consisting of D-glucuronic acid and N-acetylglucosamine, is responsible the CD44 receptor, found on the surface of cells and particularly overexpressed in certain colon cancer types^51,52^. Its overexpression is greatest in cancer stem-like cells, which are strongly related to drug resistance and metastasis^53^. Its density is higher with the stage of the cancer^54^. Moreover, in tumors, this receptor is frequently expressed in the variant of higher molecular weight isoforms, altering cell behavior and signal pathways^51^. However, in normal cells, it is found in a resting state and cannot bind to hyaluronic acid^52^. This compound is widely used in CD44-targeted therapies, for example, to ameliorate water solubility or break drug resistance^51^.

Wu et al. (2020) observed that chondroitin sulfate obtained from sturgeon (*Acipenser*) inhibited the proliferation of human colon carcinoma HCT-116 cells and induced their apoptosis^55^. It also suppressed xenograft HCT-116 tumor growth in mice by reducing proliferation and inducing apoptosis. In turn, in a study by Wu et al. (2021), chondroitin sulfate from sturgeon reduced the viability of five colorectal cancer cell lines, including Caco-2, in a concentration-dependent manner, and normal colon cells NCM460 were much less susceptible to its effects^56^. This compound also increased the proportion of HT-29 cancer cells in the G0/G1 phase of the cell cycle without affecting the cell cycle of NCM460 cells. Moreover, it induced apoptosis in HT-29 cells at a much higher rate than in normal cells. In HT-29 cells, it also affected the expression levels of 187 proliferation-related genes (among others, the expression of genes responsible for DNA replication, cell cycle, apoptosis, and PI3K-AKT pathways) and 10 proteins (involved in the transcription and translation, cell cycle, protein metabolism, apoptosis, and PI3K-AKT pathway), including the increase in BAD (Bcl2-associated agonist of cell death) and HSP (heat shock protein) 27 pro-apoptotic proteins. In our study, hydrolyzates from snail eggs tended to elevate the concentration of these proteins in Caco-2 cells. In turn, in a Wu et al. (2021) study, chondroitin sulfate reduced HT-29 transplanted tumor mice mortality, tumor volume, and histological damage and attenuated inflammatory cell recruitment into different organs. It also contributed to maintaining the proper depth of colon crypts. This compound inhibited tumor xenograft mass, necrosis, and cell infiltration. Its administration restored serum levels of pro-inflammatory cytokines and anti-inflammatory cytokine to near-normal levels. Chondroitin sulfate regulated the expression of genes responsible for colorectal cancer cell proliferation and cell cycle *in vivo*, including the increase in expression of the cyclin-dependent kinase inhibitor 1 (p21) gene. It can also activate apoptosis, and its induction was confirmed *in vivo* by Wu et al. (2021), including increased level of BAD gene expression. Our hydrolyzates from snail eggs tended to increase the level of p21 protein in Caco-2 cells. In another study by Wu et al. (2022a) on mice with HT-29 transplanted tumor, chondroitin sulfate supplementation could have significantly reduced mice mortality, suggesting that it could slow colorectal cancer development^48^. Reduced tumor biomarkers’ expression levels, related to cancer differentiation, invasion, and metastasis, also confirmed its anticancer activity. In addition, chondroitin sulfate reduced the depth of colonic crypts, its histopathological changes and inflammatory cell infiltration. Thus, it could maintain the intestinal mucosa barrier and its structural integrity, maintaining the balance of the intestinal microflora. In other research, chondroitin sulfate, at concentrations of 0.08-50 mg/mL, reduced the viability of HCT-116 cells, after 24, 48 and 72 h of treatment^57^. In addition, this compound, at a concentration of 0.08 mg/mL, reduced the mRNA expression levels of cyclin-dependent kinases (CDKs) 1, 2, 4 and 6 in HCT-116 and Caco-2 cells, after 24 h of incubation. It also significantly reduced CDK1 and CDK4 protein levels, and affected MAP (mitogen-activated protein) kinase pathways in HCT-116 cells. Moreover, it induced apoptosis of these cells, increased the number of dead cells, reduced migration and invasion. In turn, Wu et al. (2022b) showed that the hydrolyzate from sturgeon cartilage and its three fractions exhibited high *in vitro* absorption, which was inversely proportional to their molecular weight^25^. The hydrolyzate and its fractions reduced the viability of human colon cancer HT-29 cells after 24 h of incubation, and this effect was dose-dependent. Moreover, a negative effect of the hydrolyzate and two fractions on the viability of human normal colonic epithelial NCM460 cells was also found, but this effect was weaker than on cancer cells. In turn, in studies on mice, it was confirmed that fraction with the best effect on HT-29 cells and no effect on healthy cells was efficiently absorbed and transported to the tumor, significantly inhibited the growth of HT-29 cell xenograft, in a dose-dependent manner. It likely inhibited tumor cell differentiation and invasion and reduced necrosis, pyknosis, and tumor node metastasis. It suppresses the proliferation of cancer cells and activates apoptosis, through regulation of the p53 pathway and related genes, with no negative effects on healthy tissues. p53 can suppress proliferation by suppressing the growth factor signaling pathway (activates p21). In our study, there was a tendency for hydrolyzates to increase the expression of not only p21 protein but also p53 protein in Caco-2 cells. In turn, Augustyniak and McMahon (2023) confirmed the beneficial effects of chondroitin disaccharide Δdi-4 S sodium salt, of marine origin, on normal cells; it stimulated (at a concentration of 0.001-0.1 mg/mL) the proliferation of dermal fibroblasts and dermal papilla cells, as well as the production of extracellular matrix components^58^. It is worth noting that many chemotherapeutics based on glycosaminoglycans and mimetics have been creating promising results in animal models and clinical trials^55^.

In egg hydrolyzates in our experiment, the allantoin content was higher than in non-digested extracts, indicating its release from cells during hydrolysis. This compound is produced from uric acid by enzymatic or chemical oxidation^59^. It is formed by cellular metabolism in many organisms, from bacteria to vertebrates. It has long been applied in medicine due to its lack of toxicity and side effects^60^. Its therapeutic effects include stimulating cell growth, tissue regeneration, and the reconstruction of granulation tissue^59^. Allantoin stimulates fibroblast proliferation, collagen and elastin synthesis, and inhibits inflammatory cell migration. In addition, it has been reported to be beneficial in treating gastritis, has antihypertensive properties, and demonstrates hypoglycemic effects. Our hydrolyzates contained less allantoin (referring to dry matter) compared to water extracts of *H. a. aspersa* and *H. pomatia* mucus obtained using ultrasounds^59^ and more than *H. a. aspersa* mucus in the studies by El Mubarak et al. (2013)^60^ and Pagano et al. (2024)^61^. Its concentration was similar to the content in *H. a. aspersa* mucus in Di Filippo et al. (2020)^62^ and smaller than in *H. a. aspersa* mucus in Gugliandolo et al. (2021)^63^. In a Hu et al. (2024) study, blood allantoin concentration was negatively associated with oral cancer risk, and it may show protective effects against this and other cancers^64^. Its level could increase under stressful conditions and it has shown cytotoxic effects against different age-related cancers, mainly in an environment characterized by oxidative stress. In turn, in a study by Binhudayb et al. (2022), allantoin increased the apoptotic effect of 5-fluorouracil (5-FU) on Hep2 cells in a dose-dependent manner by reducing cancer stem cell markers^65^. At the same time, it showed no toxic action on normal dental pulp stem cells and oral epithelial cells. It also showed toxic effects on an *in vivo* tongue squamous cell carcinoma model, accompanied by inhibition of trans-differentiation of tumor-associated macrophages and cancer-associated fibroblasts. Marzook et al. (2021) reported that allantoin reduced the viability of colon, intestine, throat, breast, ovarian, and prostate cancer cells^66^. In turn, in a study by Parveen et al. (2017), DL-allantoin derived from *Garcinia nervosa* leaves inhibited the viability of two breast cancer cell lines in a dose-dependent manner^67^.

Snail egg hydrolyzates also contained glycolic acid, a commonly applied alpha-hydroxy acid in skin care products^60^. It stimulates collagen synthesis and accelerates cell turnover by supporting the exfoliation process^61^. Its content in our hydrolyzates was lower than in *H. a. aspersa* mucus, obtained by some authors^60,63^ and similar to the content in *H. a. aspersa* mucus described by other authors^62^. In a study by Yang et al. (2004), the administration of glycolic acid caused morphological changes in human leukemia cell line HL-60, as well as a decrease in their viability, cell cycle arrest in the G2/M phase, and induction of apoptosis in a dose- and time-dependent manner. It increased the activity of caspase-3 and − 9, but not caspase-8^68^. In turn, in the study by Ujlaki et al. (2023), glycolic acid, administered at a concentration corresponding to its concentration in human serum, showed a cytostatic effect against 4T1 murine breast cancer cells^69^.

In our research, incubation with *H. a. maxima* egg hydrolyzate at a dilution of 10^−2^ for 24 h reduced the plasma membrane integrity of Caco-2 cells. On the other hand, after 72 h, such an effect was shown for concentrated hydrolyzates from *H. a. maxima* and *H. a. aspersa* eggs and hydrolyzate from *H. a. maxima* eggs at a dilution of 10^−3^. The increased release of LDH after this incubation time with *H. a. maxima* hydrolyzate at the highest and lowest concentrations, and the lack of significant effect of the two intermediate concentrations, was most likely due to the hormesis effect^70–74^. Extreme concentrations of the hydrolyzate may have induced different mechanisms of cytotoxicity. Concentrated hydrolyzate probably contained many bioactive peptides and metabolites, leading to cell membrane damage, increasing its permeability. Medium concentrations may have had a protective or neutralizing effect, for example, through activation of antioxidant pathways or induction of detoxifying enzymes^73,74^. Such an adaptive response reduced the sensitivity of cells to stress and consequently decreased cytotoxicity. In contrast, at the lowest concentrations, certain bioactive molecules, such as peptides, lipids, and lectins, may act as prooxidants to activate ROS pathways, ligands of stress receptors such as TLRs (toll-like receptors), initiate specific pro-inflammatory signals or endoplasmic reticulum stress pathways, leading to increased cellular stress and apoptosis. Determining the mechanisms of action of the lowest concentrations of hydrolyzates, however, is beyond the scope of this paper. It is noteworthy that the concentrations of hydrolyzates effective against Caco-2 cells (referring to a weight of lyophilized eggs) were within the range of concentrations effective against various colorectal cancer cell lines of extracts of plant, fungal, and invertebrate origin^75–78^. Only the lowest concentrations of hydrolyzates, however, were within the range of concentrations effective against colorectal cancer cells of the popular chemotherapeutic agent 5-FU^79,80^. In turn, incubation of normal IEC-6 cells with concentrated *H. a. maxima* egg hydrolyzate for 24 h improved the plasma membrane integrity of these cells. These results indicate a selective effect of digested egg compounds of the studied snail subspecies on the integrity of the plasma membrane of tumor cells.

Then, treatment of Caco-2 cells with hydrolyzates for 24 and 72 h did not affect their ROS content. This may indicate that the chemical compounds in snail eggs, when digested under gastrointestinal conditions, do not generate oxidative stress in cancer cells but also in healthy cells. Therefore, the reduction of the plasma membrane integrity of Caco-2 cells by these compounds was ROS-independent. In turn, in the research of Petsantad et al. (2020), two synthetic antioxidant peptides of low molecular weight, corresponding to those contained in the hydrolyzate of defatted *Babylonia areolata* snail, obtained using pepsin and pancreatin, even limited ROS production by the Caco-2 cell monolayer, after 72 h of incubation, most likely due to the presence of hydrophobic amino acids in peptide sequences^22^.

Moreover, treatment of Caco-2 cells with hydrolyzate from *H. a. maxima* eggs for 72 h resulted in a significant reduction in their mitochondrial membrane potential, while a tendency was noted for hydrolyzate from *H. a. aspersa* eggs, indicating induction of apoptosis, programmed cell death, by snail egg compounds after digestion^19^. Loss of the intrinsic transmembrane potential of mitochondria is one of the most characteristic processes of apoptosis early stage.

We then found that incubation of Caco-2 cells with egg hydrolyzates for 72 h altered the concentration of certain apoptotic proteins in these cells, indicating activation by digested snail egg compounds of both apoptosis signaling pathways, intrinsic and extrinsic^81^. In the case of the intrinsic pathway, signals include extracellular and developmental stimuli to induce permeabilization of the outer mitochondrial membrane, release of apoptotic proteins, and activation of caspases, while in the case of the extrinsic pathway, apoptosis begins with death receptors stimulation. Moreover, some proteins whose concentration in Caco-2 cells was altered by hydrolyzate treatment, important members of the Bcl-2 (B-cell lymphoma-2) family, are apoptosis key regulators and critical intracellular checkpoints^81^. We observed an increased level of anti-apoptotic member, multi-domain protein Bcl-W, in Caco-2 cells when treated with egg hydrolyzates, while the concentration of pro-apoptotic members, containing a BH3-only group, BID and BIM, was higher in Caco-2 cells incubated with our hydrolyzates (BIM protein in the case of *H. a. maxima* hydrolyzate)^81^. It was proved that BH3-only proteins can directly activate pro-apoptotic proteins or neutralize anti-apoptotic and release pro-apoptotic proteins. Truncated BID (tBID)/BIM are the BH3-only proteins capable of binding and antagonizing the action of all Bcl-2 proteins with anti-apoptotic properties, with equal affinity, making them considered the most powerful effectors of apoptosis.

BID is the main link between extrinsic and intrinsic apoptosis^82^. It is activated by caspases, especially caspase-8, to tBID, which binds to other Bcl-2-like proteins, triggering apoptosis. tBID translocates to the outer mitochondrial membrane and promotes the pro-apoptotic proteins BAX (apoptosis regulator BAX) and BAK (BCL2 antagonist) oligomerization. Then, homo-oligomers BAX and BAK are inserted into the outer mitochondrial membrane and induce its permeabilization, followed by the leakage of proteins into the cytosol, such as cytochrome C. We noted a tendency for its lower concentration in Caco-2 cells after treatment with hydrolyzates from snail eggs, which indicates its release into the cytosol and then outside the cells due to the reduced integrity of their cell membrane. Then, cytochrome C activates caspases, which leads to cell death^82^. It is also known that tumor cells with accumulated tBID in the mitochondria are “primed” for apoptosis but may resist it due to the connection of tBID with anti-apoptotic proteins. tBID can also induce permeability of the outer mitochondrial membrane on its own. Moreover, BID activation was also observed in mitochondrial apoptosis in mitotic arrest.

BIM is also involved in the initiation of the intrinsic apoptosis pathway^81^. It was confirmed that its decreased expression is related to tumor promotion. At the same time, its overexpression contributes to the inhibition of drug resistance and tumor growth. The control of BIM expression may be used to increase the efficacy of chemotherapy as many chemotherapeutics interact with this protein. It is also worth noting that BH3 inhibitors and mimetics are being tested in clinical trials, mainly for therapies to overcome chemoresistance^82^.

The death receptor apoptosis pathway, which differs from the mitochondrial pathway, is engaged when death receptors found on the surface of certain cells bind to adapter proteins containing the death domain^83^. Receptors for death ligands belong to the TNFR (TNF receptor) family, and the principal death receptors are TRAIL, DR3, DR6, TNFR1 (tumor necrosis factor receptor 1), and CD95 (cluster of differentiation 95, FAS - apoptosis-mediating surface antigen FAS, APO-1 - apoptosis antigen 1). In our study, incubation of Caco-2 cells with *H. a. maxima* egg hydrolyzate resulted in greater production of DR6 protein in Caco-2 cells, while with *H. a. aspersa* - TRAIL-R2 and TRAIL-R3. Then, when death receptors become ligated, they activate caspase-8, and the mitochondrial apoptosis pathway is engaged^83^. Moreover, when the permeability of the outer mitochondrial membrane is triggered by tBID, antagonists of XIAP (E3 ubiquitin-protein ligase XIAP), a protein that blocks the activity of caspases-3, −7, and − 9, such as SMAC, are released from the intermembrane space of mitochondria and interfere with XIAP function, leading to the progression of apoptosis. In our research, incubation of Caco-2 cells with hydrolyzate from *H. a. aspersa* eggs resulted in a tendency for increased production of SMAC protein by these cells.

Apoptosis in cells with the TRAIL receptors can be induced by TRAIL, the TNF-family ligand, produced by T lymphocytes, among others^83^. However, not all cells with receptors for this protein are sensitive to apoptosis it induces. Then, TRAIL receptor ligation recruits FADD (FAS-associated death domain protein) to the death domain in the intracellular region, which binds and dimerizes caspase-8 for activation. In addition, TRAIL-R2 gene expression can be induced independently of TRAIL by endoplasmic reticulum stress, causing spontaneous oligomerization of TRAIL-R2 protein, leading to activation of caspase-8. Moreover, TRAIL receptors could also engage apoptosis by activating JNK (c-Jun N-terminal kinase), which can phosphorylate the BIM protein, promoting its activity. However, how caspase-8 activates JNK and BIM is currently unknown.

**Fig. 7:**
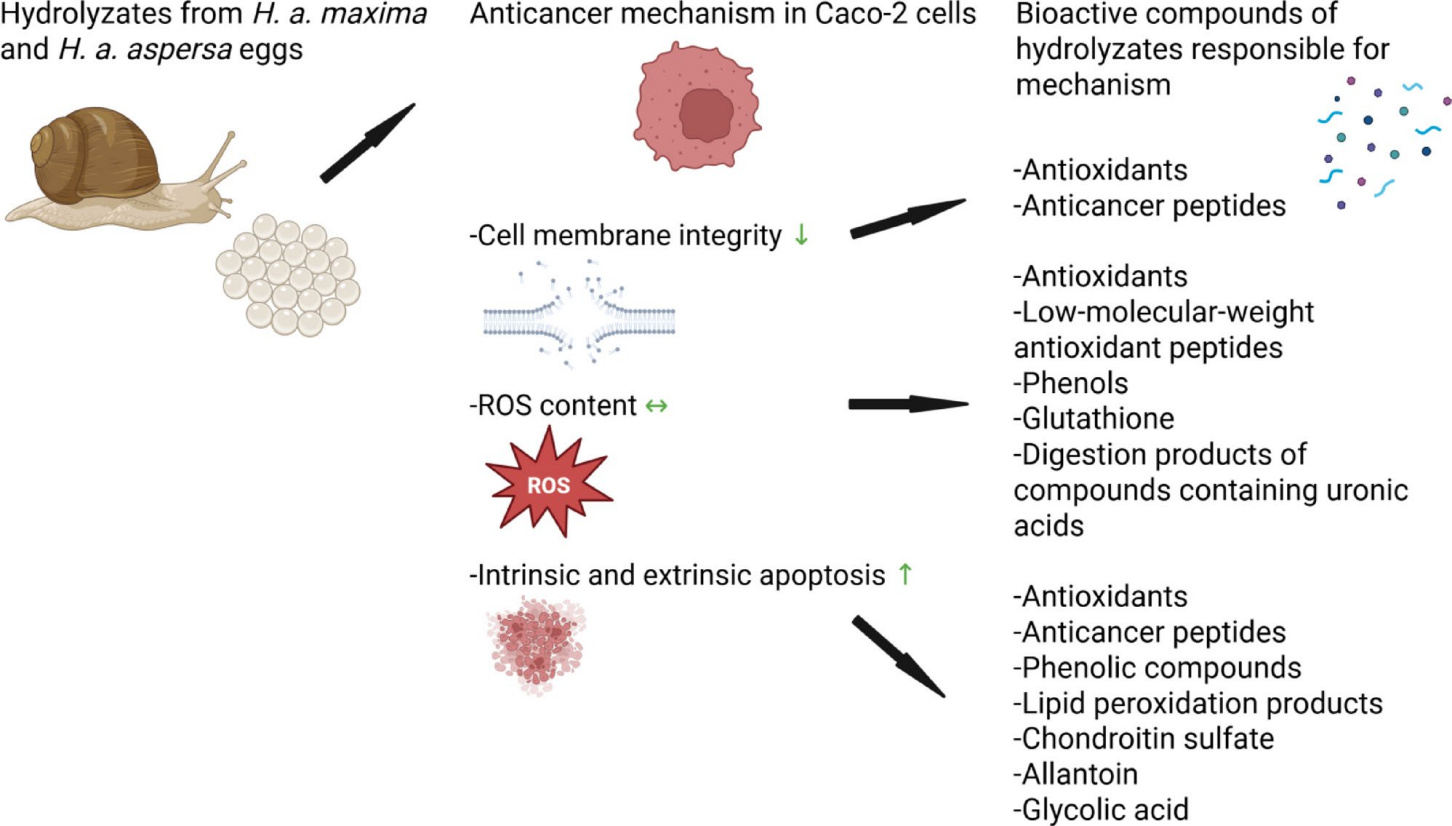
The anticancer mechanisms of action of hydrolyzates from *Helix aspersa maxima* and *Helix aspersa aspersa* eggs on Caco-2 cells, as well as the bioactive compounds of the hydrolyzates that act according to these mechanisms. Up arrow - increase, horizontal arrow - no change, down arrow - decrease. Created with BioRender.com.

The effects of hydrolyzates from *H. a. maxima* and *H. a. aspersa* eggs on the plasma membrane integrity, ROS content, and the activation of apoptosis in Caco-2 cells may be attributed to their antioxidants (Fig. 7). The low-molecular-weight antioxidant peptides of the hydrolyzates, especially < 3 kDa, may have contributed significantly to the lack of increase in ROS concentration in these cells. Other compounds, such as phenols, GSH, or the digestion products of compounds containing uronic acids, may also have been responsible for such effects. Decreased plasma membrane integrity of Caco-2 cells may be assigned to the selective action of anticancer peptides against cancer cells, which requires subsequent research. These peptides likely showed the pro-apoptotic effect. The activities of phenols, lipid peroxidation products, chondroitin sulfate, allantoin, and glycolic acid may have also contributed to apoptosis induction. The glycoprotein agglutinin and hyaluronic acid may have bound with the surface of Caco-2 cells, facilitating the delivery of anticancer compounds. Other bioactive compounds of the hydrolyzates and their additive and synergistic action most likely also showed toxic effects on Caco-2 cells.

## Methods

### Animal material and generation of hydrolyzates

0.5 kg of cocoons of two-day-old eggs of *Helix aspersa maxima* Taylor, 1883 (*Cornu aspersum maxima* (Taylor, 1883)) and *Helix aspersa aspersa* Müller, 1774 (*Cornu aspersum aspersum* (Müller, 1774)) snails were purchased from the snail breeding near Grudziądz (Poland). Raw materials were harvested in May 2020. A few hours after collection, the eggs were washed under running water, homogenized, frozen (−80 °C), lyophilized, milled, and stored (−80 °C), as before^2^.

The static *in vitro* simulation of gastrointestinal food digestion standardized protocol was used to obtain hydrolyzates from lyophilized and ground *H. a. maxima* and *H. a. aspersa* eggs^16^. Pepsin activity in commercial porcine pepsin (1639.1 U/mg; Sigma-Aldrich, St. Louis, MO, USA, P7012) and trypsin activity in porcine pancreatin (6 U/mg; Sigma-Aldrich, St. Louis, MO, USA, P1750) were determined experimentally, using standardized assays^16^. The concentration of bile salts in bovine bile (1,44 mmol/g; Sigma-Aldrich, St. Louis, MO, USA, B3883) was based on literature data^84^; this content differed slightly in products from different manufacturers. Simulated digestive fluids: salivary (pH 7), gastric (pH 3), and intestinal (pH 7) were prepared^16^. Immediately before each digestion phase, deionized water, 0.3 M CaCl_2_(H_2_O)_2,_ and digestive fluids were heated (37 °C). Enzyme solutions (36,6 mg pepsin/mL deionized water and 133.3 mg pancreatin/mL simulated intestinal fluid) and bile solution (92.6 mg/mL simulated intestinal fluid) were prepared. 1 g of lyophilized *H. a. maxima* or *H. a. aspersa* eggs was weighed into 50 mL polypropylene tubes, and 4 mL of deionized water was added. Two samples were prepared for each snail subspecies, where the hydrolyzate was animal material treated with digestive fluids and enzymes. The non-digested extract was animal material that was exposed to digestive fluids without the addition of enzymes. A blank sample was prepared by adding digestive fluids with enzymes to deionized water (1 mL) instead of eggs. The digestion procedure started with the oral phase. 4 mL of simulated salivary fluid and 25 µl of 0.3 M CaCl_2_(H_2_O)_2_ were added to all samples. Salivary amylase was not used, the application of which is only recommended for starch-containing foods. The samples were supplemented with 0.975 mL of deionized water. Then, the second phase of digestion was carried out - the gastric phase. 8 mL of simulated gastric fluid was added to all samples. 0.450 mL of concentrated HCl (35–38%) was introduced into the samples containing snail eggs, and 10 µl of concentrated HCl was added to the blank sample, which allowed the pH to be lowered to 3.0. 5 µl of 0.3 M CaCl_2_(H_2_O)_2_ was added to all samples. A 0.667 mL pepsin solution (2000 U/mL final digestion mixture) was added to the hydrolyzed and blank samples. Gastric lipase was not used because snail eggs contain practically no fat^2^; lipase with appropriate activity is also difficult to access commercially^16^. Hydrolyzed samples were supplemented with 0.878 mL of deionized water, non-digested samples − 1.545 mL, and the blank sample − 1.318 mL. Samples were incubated and mixed for two hours using a rotator (vertical rotation speed 30 rpm; Multi Bio RS-24; Biosan, Riga, Latvia) and placed in an incubator (37 °C; Stuart, Stone, UK). The starting time of incubation was the moment of adding the pepsin solution. The third phase of the digestion procedure was the intestinal phase, which was initiated by introducing 8 mL of simulated intestinal fluid to all samples. Then, the pH was adjusted to 7.0 by adding 0.035 mL of 5 M NaOH to the samples containing eggs and 0.11 mL of concentrated HCl to the blank sample. 3 mL of bile solution (10 mM in the final mixture) and 40 µl of 0.3 M CaCl_2_(H_2_O)_2_ were added to all samples. Pancreatin solution (5 mL) containing trypsin (activity of 100 U/mL final mixture) and lipase were introduced into the hydrolyzed and blank samples. 5 mL of simulated intestinal fluid was added to the non-digested samples. The samples were supplemented with deionized water, containing eggs – 3.925 mL and blank sample − 3.850 mL. Samples were incubated (37 °C) and mixed for two hours as before. The last stage of the procedure was the inactivation of enzymes; for this purpose, the samples were placed in a water bath (95 °C, 5 min.). Then, to separate the bioaccessible phase from unavailable compounds, the samples were centrifuged three times (5000 rpm, 10 min) and once (14000× *g*, 10 min). The resulting supernatants were diluted four times with deionized water and filtered through filter paper into new tubes. The vortexed supernatants were then divided into 1.5 mL tubes for further analysis, frozen in liquid nitrogen, and stored (−80 °C).

### Determination of redox status indicators

Ferric-reducing antioxidant power in appropriately diluted with deionized water, hydrolyzates, and non-digested extracts from *H. a. maxima* and *H. a. aspersa* eggs and digestive fluids was assessed using the modified Oyaizu procedure^85^, described in English in an article by Sun et al. (2007)^86^, exactly as in our previous article (our modification consisted of using twice the proportion of 0.1% ferric chloride relative to the test samples and using a standard curve)^2^. *n* (number of replicates) = 6.

DPPH· scavenging activity in the above undiluted samples was determined as in Matusiewicz et al. (2022)^2^. The method we use differs from the original procedure of Brand-Williams et al. (1994)^87^ in the proportion of test samples to reagent, the concentration of reagent, the presence of a centrifugation step, the use of a standard curve, and the measurement at the absorption maximum, *n* = 6.

ABTS·^+^ scavenging activity in appropriately diluted samples was evaluated as in Matusiewicz et al. (2022)^2 ^with the ABTS reagent diluted with deionized water instead of absolute ethanol. A kinetic reading was carried out for 180 min. Our method differs from the original method of Fellegrini et al. (1999)^88^ by preparing standards in deionized water, *n* = 6.

The content of total phenols in undiluted samples was analyzed by applying the Folin-Ciocalteu procedure^89^ as in Matusiewicz et al. (2022)^2^. Gallic acid was used to construct the standard curve, *n* = 6.

GSH concentration was determined using the Ellman method^90^ as in Matusiewicz et al. (2022)^2^. In our procedure, 2-nitrobenzoic acid was dissolved in 99.9% ethanol, and a standard curve was prepared, *n* = 6.

TBARS content was measured using the Uchiyama and Mihara method^91^ as in Matusiewicz et al. (2022)^2^. Into 100 µL of test samples and standards (0–100 nmol/mL 1.2.3.3-tetraethoxypropane) were introduced 400 µL 154 mM KCl, 25 µL 2% butylated hydroxyanisole in 99.9% ethanol, 1.5 mL 1% H_3_PO_4_, and 0.5 mL 30 mM thiobarbituric acid. After vortexing, the samples were incubated in a water bath (95 ºC, 1 h) and cooled. Then, 2 mL of 99% 1-butanol was added, and the samples were vortexed. After centrifugation (5000 rpm, 15 min), absorbance in the butanol phase was measured as in Matusiewicz et al. (2022)^2^. Our procedure differs from the original one in the volume and concentration of reagents, a larger number of reagents, and measurement at the absorption maximum for the TBARS complex, *n* = 6.

The level of protein carbonyls was analyzed by applying the method of Levine et al. (1990)^92^ as in Matusiewicz et al. (2023)^93^; vortexing was used to dissolve trichloroacetic acid (TCA) pellets, *n* = 3.

### Determination of total protein and total carbohydrates

The total protein level in hydrolyzates, non-digested extracts, and digestive fluids was determined using the Bradford method^2^, *n* = 6.

The content of total carbohydrates in the appropriately diluted samples above was assessed by the phenol-sulfuric acid procedure^2^, *n* = 3.

### SDS-PAGE profile of proteins and glycoproteins

Hydrolyzates, non-digested extracts, and digestive fluids were subjected to SDS-PAGE as in Matusiewicz et al. (2022)^2^. Two 10% resolving gels were applied to separate proteins and glycoproteins using the Laemmli procedure with modifications. Each sample was submitted to denaturation and reduction by mixing in a 1:1 ratio with the Laemmli sample buffer with β-mercaptoethanol and heating. 20 µL of samples and 5 µL of protein marker (ColorBurst^™^ Electrophoresis Marker; Sigma-Aldrich, St. Louis, MO, USA) were applied onto the gels and resolved. 10 µL of the positive control, horseradish peroxidase (2 mg/mL), mixed with Laemmli sample buffer, was also loaded onto the gel to prepare the glycoprotein profile.

The fixed proteins on the first gel were stained with QC Colloidal Coomassie Stain (Bio-Rad Laboratories, Hercules, CA, USA). The fixed glycoproteins on the second gel were stained using the periodic acid-Schiff method.

ImageJ software (the National Institutes of Health and the Laboratory for Optical and Computational Instrumentation (the University of Wisconsin, WI, USA)) was applied for densitometric (integrated density) analysis of gel photos. The lower, unseparated band of ~ 8 kDa was not included in the densitometric analysis; additionally, for glycoproteins, it was measured separately.

### Measurement of uronic acids, allantoin, and glycolic acid

The reaction of uronic acids with carbazole allows the determination of the content of polysaccharides and proteoglycans extracted from various tissues^94^. 50 µL of diluted hydrolyzates, non-digested extracts, and digestive fluids were mixed with 200 µL of 25 mM sodium tetraborate in sulfuric acid. The samples were incubated in a water bath (95 °C, 10 min) and then cooled to room temperature (15 min). The samples were mixed with 50 µL of 0.125% carbazole in 99.9% ethanol, incubated in a water bath, and cooled. Samples were vortexed and transferred to a 96-well plate for absorbance reading in a microplate reader (Infinite M200 microplate reader; Tecan, Männedorf, Switzerland) at a wavelength of 550 nm. Hyaluronic acid (0–1 mg/mL) was used as a standard, *n* = 6.

In the method used to determine allantoin, it is hydrolyzed to allantoic acid, which is hydrolyzed to glyoxylic acid^95^. Glyoxylic acid hydrazone is formed and then converted to a chromophore. 156 µL of hydrolyzates, non-digested extracts, and digestive fluids were mixed with 31 µL of 0.6 M NaOH and then incubated in a water bath (95 °C, 12 min). Then, the samples were mixed with 63 µL of 0.1% 2,4-dinitrophenylhydrazine in 2 M HCl and incubated in a water bath (95 °C, 3 min). The samples were cooled to room temperature and mixed with 313 µL of 2.5 M NaOH. After 10 min, the samples were placed on a 96-well plate, and the absorbance was read in a microplate reader at a wavelength of 520 nm. Allantoin (3.9–125 µg/mL) was used as a standard, *n* = 5.

The concentration of glycolic acid in hydrolyzates, non-digested extracts, and digestive fluids was determined using the colorimetric method^96^. 100 µL of the above samples were mixed with 100 µL of 5% TCA and then centrifuged (3000× *g*, 5 min). Then, 50 µL of supernatants were mixed with 500 µL of chromotropic acid (2 mg chromotropic acid disodium salt/1 mL concentrated H_2_SO_4_) and placed in a water bath (95 °C, 10 min). After cooling, the samples were mixed with 4.5 mL of deionized water and put on a 96-well plate. The absorbance was read (microplate reader) at a wavelength of 580 nm. Glycolic acid (0-1.2 mg/mL) was used as a standard, *n* = 4.

### Preparation of hydrolyzates for tests on cell cultures

Hydrolyzates and non-digested extracts from *H. a. maxima* and *H. a. aspersa* eggs and digestive fluids were sterilized (polyvinylidene fluoride (PVDF) syringe filters, pore diameter 0.22 μm; Merck Millipore, Burlington, MA, USA) under a biological safety cabinet (Airstream®, class II; Esco, St. Louis, MI, USA). For the test described in the “Effect of hydrolyzates on the integrity of plasma membranes of Caco-2 and IEC-6 cells” section, 10-, 100- and 1000-fold dilutions of the above samples were prepared using sterile deionized water.

### Caco-2 and IEC-6 cell culture

A human epithelial colorectal adenocarcinoma cell line (Caco-2; the European Collection of Authenticated Cell Cultures, Sigma-Aldrich, St. Louis, MO, USA; 55 passage) and a control rat intestinal epithelial cell line (IEC-6; the American Type Culture Collection, Manassas, VA, USA; 17 passage) were cultured in 96-well polystyrene plates intended for adherent culture, with an initial concentration of 1 × 10^4^ cells/100 µL of medium, for the test described in “Effect of hydrolyzates on the integrity of plasma membranes of Caco-2 and IEC-6 cells” section. Caco-2 cells were cultured in the same medium as in Matusiewicz et al. (2022)^2 ^and Dulbecco’s Modified Eagle’s Medium (DMEM) containing 4.5 g/L glucose, 4 mM L-glutamine, 1 mM sodium pyruvate, 5% fetal bovine serum (FBS) and 1% antibiotic-antimycotic (Gibco^™^, Waltham, MA, USA) was used to culture IEC-6 cells. Caco-2 cells were cultured as above for the “Effect of hydrolyzates on the mitochondrial membrane potential of Caco-2 cells” experiment. For the “Influence of hydrolyzates on the content of ROS in Caco-2 cells” test, Caco-2 cells were cultured in 24-well plates (initial density: 5.94 × 10^4^ cells/594 µL), and for the “Influence of hydrolyzates on the production of apoptotic proteins by Caco-2 cells” experiment – in 6-well plates (initial density: 0.75 × 10^5^ cells/1.5 mL). The cells were placed in a CO_2_ incubator (37 °C, 5% CO_2_, 95% relative humidity)^2^, Caco-2 cells - for 24 h, until approximately 70% confluence was achieved, and IEC-6 cells - for 48 h, until a monolayer was formed. After this time, the cells were starved overnight in a medium with 1% FBS content, and in the case of Caco-2 cells - without adding non-essential amino acids (NEAA).

### Effect of hydrolyzates on the integrity of plasma membranes of Caco-2 and IEC-6 cells

The release of the cytosolic enzyme lactate dehydrogenase (LDH) into the medium, associated with changes in cell membrane permeability, was determined as previously^2^. 100 µL of fresh medium and 10 µL of hydrolyzates and non-digested extracts from *H. a. maxima* and *H. a. aspersa* eggs, concentrated and diluted 10, 100, and 1000 times, as well as 10 µL of digestive fluids were introduced into the cells. Additional control cells were included to determine spontaneous LDH activity (after the addition of 10 µl of sterile deionized water) and maximum LDH activity (after cell lysis with lysis buffer). After 24 and 72 h of incubation (CO_2_ incubator), LDH activity was determined and expressed as $\:\frac{\text{c}\text{o}\text{m}\text{p}\text{o}\text{u}\text{n}\text{d}-\text{t}\text{r}\text{e}\text{a}\text{t}\text{e}\text{d}\:\text{L}\text{D}\text{H}\:\text{a}\text{c}\text{t}\text{i}\text{v}\text{i}\text{t}\text{y}-\text{s}\text{p}\text{o}\text{n}\text{t}\text{a}\text{n}\text{e}\text{o}\text{u}\text{s}\:\text{L}\text{D}\text{H}\:\text{a}\text{c}\text{t}\text{i}\text{v}\text{i}\text{t}\text{y}{\phantom{^{1^{1}}}}}{\text{m}\text{a}\text{x}\text{i}\text{m}\text{u}\text{m}\:\text{L}\text{D}\text{H}\:\text{a}\text{c}\text{t}\text{i}\text{v}\text{i}\text{t}\text{y}-\text{s}\text{p}\text{o}\text{n}\text{t}\text{a}\text{n}\text{e}\text{o}\text{u}\text{s}\:\text{L}\text{D}\text{H}\:\text{a}\text{c}\text{t}\text{i}\text{v}\text{i}\text{t}\text{y}}\:\times\:100$, *n* = 6.

### Influence of hydrolyzates on the content of ROS in Caco-2 cells

The level of ROS in Caco-2 cells was determined using DCFH-DA (2’,7’-dichlorodihydrofluorescein diacetate), which is taken up by cells in which esterase cleaves acetyl groups to form DCFH (2’,7’-dichlorodihydrofluorescein). Then, under the influence of ROS, it is oxidized to DCF (2’,7’-dichlorofluorescein), with green fluorescence^97^. 535 µL of new medium and 59 µL of hydrolyzates, non-digested extracts, digestive fluids, or deionized water were introduced into the plates. After 24 and 72 h of incubation (CO_2_ incubator), the medium was withdrawn, and the cells were washed with MEM. Then, 500 µL of 10 µM DCFH-DA in MEM (prepared from 10 mM stock solution in dimethyl sulfoxide (DMSO)) was added. The plates were placed in a CO_2_ incubator for 30 min. The solution was withdrawn, and the cells were washed with MEM followed by PBS (phosphate buffered saline), pH 7.4. Then, to lyse the cells, 200 µl of RIPA (radioimmunoprecipitation assay) buffer was introduced into the wells, and the cells were incubated at 4 °C (5 min). Then, the lysates were collected into Eppendorf tubes and centrifuged (21130× *g*, 10 min, 4 °C). 150 µl of supernatants were transferred to a black 96-well glass-bottom plate, and fluorescence was measured at an excitation wavelength of 485 nm and an emission wavelength of 530 nm (microplate reader). 5 µl of supernatants were intended to measure total protein using the Bradford method^2^. The results were expressed as % of fluorescence intensity/µg total protein relative to control cells treated with deionized water. *n* = 6.

### Effect of hydrolyzates on the mitochondrial membrane potential of Caco-2 cells

The mitochondrial membrane potential of Caco-2 cells was determined using the cell membrane-permeable, fluorescent, cationic dye JC-1 (5,5′,6,6′-tetrachloro-1,1′,3,3′-tetraethyl-imidacarbocyanine iodide), with green emission (maximum at ~ 520 nm). When this dye accumulates in intact mitochondria, it forms aggregates with orange-red fluorescence (maximum at ~ 590 nm). The ratio of orange-red to green fluorescence is used as an indicator of mitochondrial membrane potential. It was determined according to the manufacturer’s procedure for the commercial kit (Cell Signaling Technology, Danvers, MA, USA). 90 µL of new medium and 10 µL of hydrolyzates, non-digested extracts, digestive fluids, and deionized water were added to the cells. After 72 h of incubation (CO_2_ incubator), 10 µL of 20 µM JC-1 in MEM (prepared by dissolving 5 mg of JC-1 from Invitrogen^™^ (Waltham, MA, USA) in 5 mL of DMSO and then diluting in MEM) was added to the wells and incubated again for 15 min (CO_2_ incubator). Then, the solution was removed from the wells, the cells were washed with PBS, and 100 µL of new PBS was introduced into them. Fluorescence was measured (excitation − 480 nm, emission − 520 nm, and 590 nm) in a microplate reader. The emission ratio at 590 nm to 520 nm was calculated. Results are expressed as % of the value of control cells incubated with deionized water. *n* = 5.

### Influence of hydrolyzates on the production of apoptotic proteins by Caco-2 cells

1.5 mL of fresh medium and 150 µL of hydrolyzates, non-digested extracts, digestive fluids, and deionized water were introduced into the wells. Each research group included cells on three plates. After 72 h of incubation (CO_2_ incubator), the procedure of the commercial kit (Human Apoptosis Antibody Array – Membrane; Abcam, Cambridge, UK) was followed. Cells were trypsinized and washed twice with cold PBS. Then, after centrifugation, the supernatants were removed, and the cells were suspended in a cold lysis buffer and transferred to four test tubes intended for different research groups. The samples were incubated for 30 min (4 °C), then centrifuged (14000× *g*, 10 min), and the supernatants were transferred to new tubes, frozen in liquid nitrogen, and stored (−80 °C). The concentration of total protein in lysates was determined by a colorimetric method based on bicinchoninic acid (BCA), using the Pierce^™^ BCA Protein Assay Kit (Thermo Fisher Scientific, Waltham, MA, USA), *n* = 4. The commercial kit’s procedure for determining apoptotic protein concentration was further followed. The total protein level in the lysates was equalized using a Blocking Buffer and amounted to 386 µg/1.2 mL (per membrane). Chemiluminescence detection on membranes was performed using the Azure c400 imaging system (Azure Biosystems, Dublin, CA, USA). ImageJ software (the National Institutes of Health and the Laboratory for Optical and Computational Instrumentation (the University of Wisconsin, WI, USA)) was used for densitometric analysis of membrane photos. The results of concentrations of apoptotic proteins were expressed relative to the results for control cells treated with deionized water. *n* = 2.

### Statistical analysis

Numerical results are presented as mean ± standard error of the mean (SEM), and in the case of bar graphs, error bars indicate SEM. The results were subjected to a one-way analysis of variance (ANOVA). The mean values of chemical composition results were compared using Tukey’s post-hoc test by applying Statgraphics Centurion XVI.I software (StatPoint Technologies, Inc., Warrenton, VA, USA). The means for the hydrolyzate and non-digested extract treated groups were compared to the digestive fluid treated group using Dunnett’s post-hoc test. Prism 5 software (GraphPad Software Inc., San Diego, CA, USA) was applied. The difference between the mean values at *p* < 0.05 was statistically significant.

## Conclusions

Bioactive compounds of *H. a. maxima* and *H. a. aspersa* snail eggs, formed after their digestion in the gastrointestinal tract, of antioxidant nature, preventing the formation and inhibiting the activity of ROS, may act beneficially in colorectal cancer initiation and progression. The reduction in the integrity of the plasma membrane, the absence of an increase in ROS level and the induction of apoptosis in Caco-2 cells under treatment with hydrolyzates from snail eggs may resulted from contained antioxidants, low-molecular-weight peptides with potentially selective effects on cancer cells, phenolic compounds, GSH, products of lipid peroxidation, compounds containing uronic acids, allantoin and glycolic acid. It is highly likely that other bioactive compounds of digested snail eggs and their additive and synergistic action also contributed to the toxic influence on Caco-2 cells. Moreover, the consumption of snail eggs, by affecting proteins of the intrinsic and extrinsic apoptosis pathway, may sensitize the response to various therapeutic strategies for colorectal cancer, which requires further research. The lack of toxic effect on normal intestinal cells also indicates the potential for using the examined snail eggs in cancer prophylaxis. Further research is needed, such as establishing a safe intake level and determining allergenicity in rodent models. *H. a. maxima* and *H. a. aspersa* snail eggs may enjoy consumer interest as a dietary supplement or novel food.

## Electronic supplementary material

Below is the link to the electronic supplementary material.

Supplementary Material 1

## Data Availability

The datasets generated during the current study are available from the corresponding author upon reasonable request.
