# Supplementary material for: Toxic effects of Helix aspersa snail egg hydrolyzates obtained by static in vitro digestion on Caco-2 colorectal adenocarcinoma cells
Source: Sci Rep. 2025 Jul 25;15:27117. doi: 10.1038/s41598-025-11605-7 (PMC12297354; doi:10.1038/s41598-025-11605-7)
Supplement: Supplementary file 1 — Supplementary Material 1 [file 41598_2025_11605_MOESM1_ESM.pdf]

Toxic effects of *Helix aspersa* snail egg hydrolyzates obtained by static *in vitro* digestion on Caco-2 colorectal adenocarcinoma cells

Magdalena Matusiewicz[1,*], Joanna Kuczka[1], Michalina Danił[1], Klara Piotrowska[2], Hanna Antushevich[3], and Tomasz Niemiec[2]

[1]Department of Nanobiotechnology, Institute of Biology, Warsaw University of Life Sciences, Warsaw, Poland
[2]Department of Animal Breeding and Nutrition, Institute of Animal Sciences, Warsaw University of Life Sciences, Warsaw, Poland
[3]Department of Genetic Engineering, The Kielanowski Institute of Animal Physiology and Nutrition, Polish Academy of Sciences, Jabłonna, Poland
[*]magdalena_matusiewicz@sggw.edu.pl

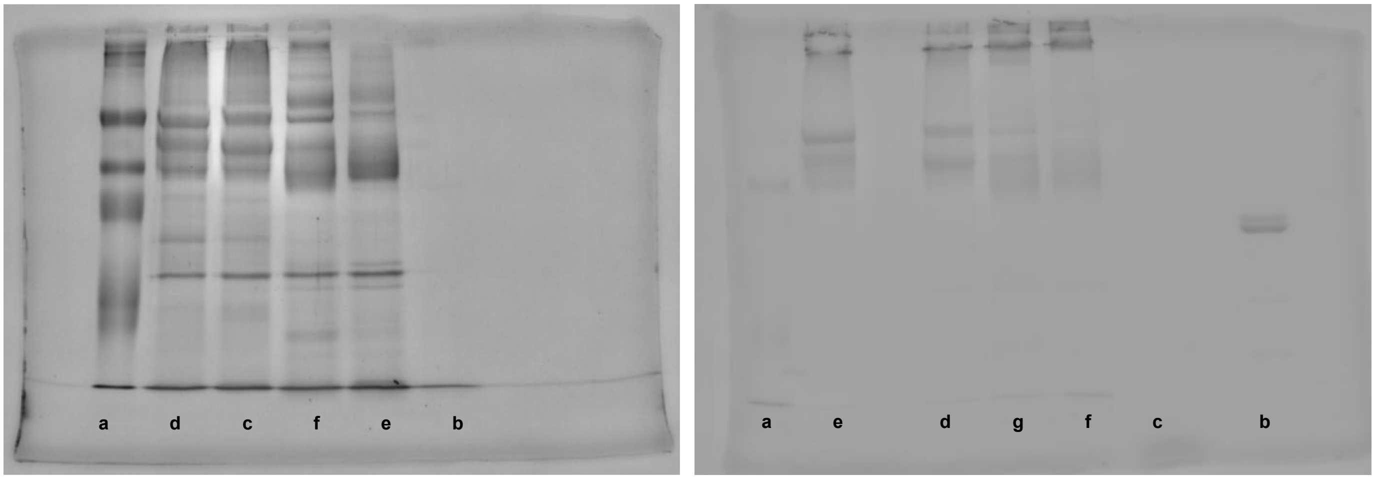

(A) (B)

Supplementary Figure S1. (**A**) Protein and (**B**) glycoprotein profile of hydrolyzates from *Helix aspersa maxima* and *Helix aspersa aspersa* eggs, obtained by SDS-PAGE. (**A**) (a) protein molecular weight marker (Sigma-Aldrich, St. Louis, MO, USA); (b) digestive fluids; (c) non-digested extract from *H. a. maxima* eggs; (d) non-digested extract from *H. a. aspersa* eggs; (e) hydrolyzate from *H. a. maxima* eggs and (f) hydrolyzate from *H. a. aspersa* eggs. (**B**) (a) protein molecular weight marker (Sigma-Aldrich, St. Louis, MO, USA); (b) horseradish peroxidase (positive control); (c) digestive fluids; (d) non-digested extract from *H. a. maxima* eggs; (e) non-digested extract from *H. a. aspersa* eggs; (f) hydrolyzate from *H. a. maxima* eggs and (g) hydrolyzate from *H. a. aspersa* eggs.

Supplementary Table S1. Densitometric (integrated density) analysis of protein and glycoprotein profile of hydrolyzates from *Helix aspersa maxima* and *Helix aspersa aspersa* eggs, obtained by SDS-PAGE (shown in Figure 3 and Supplementary Fig. S1).

**Concentration (integrated density)**	**Digestive fluids**	**Positive control (horseradish peroxidase)**	***H. a. maxima* non-digested extract**	***H. a. aspersa* non-digested extract**	***H. a. maxima* hydrolyzate**	***H. a. aspersa* hydrolyzate**
Proteins 8 – 220 kDa	5,351	-	11,764	13,056	11,652	14,097
Proteins >50 kDa	2,361	-	6,723	7,783	6,365	8,522
Proteins <20 kDa	1,505	-	1,863	1,691	2,092	2,444
Proteins >20 kDa	3,949	-	9,952	11,969	9,778	11,833
Glycoproteins 8 – 220 kDa	0,819	1,587	1,877	1,990	1,182	1,470
Glycoproteins >50 kDa	0,448	0,357	1,446	1,576	0,725	0,996
Glycoproteins 50 – 100 kDa	0,258	0,229	1,102	1,109	0,492	0,709
Glycoproteins ~8 kDa	0,056	0,073	0,081	0,090	0,164	0,139
